# Functional appliance treatment for mandibular fractures: A systematic review with meta‐analyses

**DOI:** 10.1111/joor.13178

**Published:** 2021-05-24

**Authors:** Corinne Stähli, Theodore Eliades, Spyridon N. Papageorgiou

**Affiliations:** ^1^ Clinic of Orthodontics and Pediatric Dentistry Center of Dental Medicine University of Zurich Zurich Switzerland

**Keywords:** condylar fracture, functional appliances, mandibular fracture, orthodontics, orthopaedic treatment, systematic review

## Abstract

**Objectives:**

Mandibular collum fractures among growing patients can lead to abnormal growth, function, esthetics and ultimately quality of life. Among the proposed treatment alternatives, orthopaedic treatment with functional appliances has been suggested, with encouraging results. Aim of the present systematic review was to critically appraise existing evidence on the outcome of functional appliance treatment among growing patients with mandibular collum fractures.

**Materials and methods:**

Eight databases were searched up to October 2020 for randomised and non‐randomised clinical studies assessing functional appliance treatment outcome for children with mandibular fractures. After duplicate study selection, data extraction and risk of bias assessment, random effects meta‐analyses of mean differences (MD) and their 95% confidence intervals (CIs) were performed, followed by assessment of the quality of evidence with GRADE.

**Results:**

A total of 8 unique studies (one prospective and nine retrospective non‐randomised) with 223 children could be identified. Functional appliance treatment was associated with greater anteroposterior condyle dimensions of the injured condyle compared with the contralateral healthy condyle (3 studies; MD = 0.87 mm; 95% CI = 0.30 to 1.45 mm; *p* = .003). No difference was found in the mesiodistal condyle size between the injured and the contralateral healthy joint (3 studies; MD = −0.05 mm; 95% CI = −1.05 to 0.95 mm; *p* = .92), but collum length was smaller at the injured side compared with the contralateral one (1 study; MD = −2.89 mm; 95% CI = −5.29 to −0.49 mm; *p* = .02). Treatment outcome might be influenced by patient age, patient sex and severity/localisation of the fracture, but the quality of evidence for all analyses was very low due to methodological limitations leading to bias.

**Conclusions:**

While some evidence exists that functional appliances might lead to good clinical rehabilitation of fractured mandibular condyles, including considerable bone remodelling, available studies are small and have methodological weaknesses.

## Graphical abstract

This systematic review summarizes evidence from 8 non‐randomized clinical studies (223 patients) on mandibular condyle fracture treatment outcome with functional appliances. Some data indicate that functional appliance treatment is associated with partial or complete remodelling of the fractured condyle and the temporomandibular joint with acceptable clinical results in growing patients. However, existing studies are mostly old, single‐group cohort studies without control groups and with many methodological issues and uncertainty still exists around the long‐term outcomes of functional appliances for condylar fractures.

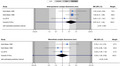

## 1 INTRODUCTION

### 1.1 Rationale

The mandibular condyle is one of the most common sites of facial skeleton that is subject to injury^1^ and, due to its role in mandibular form and function, may adversely affect growth and development of the stomatognathic system. Among growing children, acute injuries to the mandibular condyle might lead to serious adverse effects such as temporomandibular dysfunction, disturbed mandibular growth and temporomandibular joint ankylosis.^2^

Several therapeutic approaches for condylar fractures exist, including conservative treatment (observation, soft diet, analgetic use), intermaxillary fixation, functional appliance therapy, surgical treatment or a combination thereof.^2^ Particularly among children, surgical treatment might not be the first treatment of choice due to the possibility of external scars, nerve damage and abnormal post‐surgical growth.^3^, ^4^

Conservative treatment with or without intermaxillary fixation seems to often result in good mandibular function, but with the condyles not being completely remodelled in large portion of the patients and with possible late complications such as ankylosis, disturbances of facial growth or functional disorders of the temporomandibular joint.^3^, ^4^, ^5^, ^6^, ^7^

On the other hand, orthopaedic treatment with functional appliances that reposition the mandible has been reported to aid stabilisation and rehabilitation, while minimising morbidity.^8^, ^9^, ^10^ Functional appliances used for Class II malocclusion due to retrognathic mandibles have been associated with adaptations of the condyle and the glenoid fossa,^11^ and early studies in the 90s have shown encouraging results for condylar fractures—including very good remodelling rates.^8^, ^12^ However, evidence on the efficacy and safety of orthopaedic rehabilitation for collum fractures remains inconclusive.

### 1.2 Objective

Therefore, aim of this systematic review was to assess the evidence from clinical studies on humans undergoing orthopaedic treatment with functional appliances after any kind of mandibular condyle fracture.

## 2 MATERIALS AND METHODS

### 2.1 Protocol and registration

This review's protocol was made a priori and registered in Open Science Framework (https://osf.io/8ry6p/), and any deviations were noted (Supplement 1). This review is conducted and reported according to Cochrane Handbook^13^ and PRISMA statement,^14^ respectively.

### 2.2 Eligibility criteria

According to the Participants, Intervention, Comparison, Outcome, Study design (PICOS) schema and due to the scarcity of randomised clinical trials on this subject, included were randomised and non‐randomised clinical studies on human patients with growing potential (<18 years of age), of any sex, ethnicity or malocclusion with any kind of condylar fractures treated with any kind of functional appliances. No limitations concerning language, publication year or status were applied. Excluded were animal studies, case reports and non‐clinical studies. The primary outcome for this review was the restoration of function (treatment success) as reported by the patient. Secondary outcomes included dimensions of the condyle, morphology of the condyle, joint pain, joint sounds during mouth opening and midline deviations in occlusion.

### 2.3 Information sources and search

Eight electronic databases were searched systematically without any restrictions for publication date, language or type from inception up to 1 October 2020 (Supplement 2), while Directory of Open Access Journals (DOAJ), Digital Dissertations, metaRegister of Controlled Trials, WHO and Google Scholar, as well as the reference/citation lists of eligible articles or existing systematic reviews were manually searched for any additions.

### 2.4 Study selection

Two authors (CS and SNP) screened the titles and/or abstracts of studies retrieved from the searches to identify articles that potentially meet the inclusion criteria, before moving to their full texts. Any differences between the two authors were resolved by discussion with a third author (TE).

### 2.5 Data collection process and items

Data collection from the identified reports was conducted using pre‐defined and piloted forms covering: (a) study characteristics (design, clinical setting, country), (b) patient characteristics (age, sex), (c) characteristics of the mandibular fracture (affected sides, Spiessl & Schroll categorisation^15^), (d) functional appliance used, and (e) follow‐up period. Data were extracted by two authors (CS and SNP) with the same way to resolve discrepancies as above.

### 2.6 Risk of bias of individual studies

The risk of bias of included randomised studies was to be assessed according to Cochrane guidelines with the RoB 2.0 tool for randomised trials.^16^ The risk of bias of non‐randomised studies was assessed with a customised checklist based on the ROBINS‐I (‘Risk Of Bias In Non‐randomised Studies—of Interventions’) tool for non‐randomised studies.^17^ Assessment of the risk of bias of included studies was likewise performed independently by two authors (CS and SNP), with the same way to resolve discrepancies consulting a third author (TE).

### 2.7 Data synthesis and summary measures

An effort was made to include all existing trials in the analysis; where data were missing, they were calculated by ourselves or extracted from graphs (Supplement 1). As the outcome of orthopaedic treatment with functional appliances is bound to be affected by patient‐ and treatment‐related characteristics, a random‐effects model was deemed appropriate to calculate the average distribution of true effects, based on clinical and statistical reasoning,^18^ and a restricted maximum‐likelihood random‐effects model was used according to recent guidance.^19^ Mean differences (MDs) for continuous outcomes and odds ratios (ORs) for binary outcomes and their corresponding 95% confidence intervals (CIs) were calculated as effect sizes.

The extent and impact of between‐study heterogeneity was assessed by inspecting the forest plots and by calculating the τ^2^ (absolute heterogeneity) and the I^2^ statistics (relative heterogeneity; inconsistency), respectively. I^2^ defines the proportion of total variability in the result explained by heterogeneity, and not chance, and we considered arbitrarily I^2^ over 75% to represent considerable heterogeneity, while also considering the heterogeneity's direction (localisation on the forest plot) and uncertainty intervals around heterogeneity estimates.^20^ Ninety‐five per cent predictive intervals were calculated for meta‐analyses of ≥3 trials to incorporate existing heterogeneity and provide a range of possible effects for a future clinical setting, which are crucial for the correct interpretation of random‐effects meta‐analyses.^21^

### 2.8 Additional analyses and risk of bias across studies

Possible sources of heterogeneity were a priori planned to be sought through subgroup analyses and random‐effects meta‐regression in meta‐analyses of at least 5 trials but could ultimately not be performed (Supplement 1). Likewise, reporting biases were planned but ultimately not assessed, due to the limited number of meta‐analysed trials.

The overall quality of meta‐evidence (ie, the strength of clinical recommendations) was rated using the Grades of Recommendations, Assessment, Development and Evaluation (GRADE) approach^22^ following recent guidance on combining randomised with non‐randomised studies.^23^

Robustness of the results was planned a priori to be checked with sensitivity analyses based on (a) inclusion/exclusion of non‐randomised studies, (b) inclusion/exclusion of trials with methodological shortcomings and (c) improvement of the GRADE classification. In the end, no sensitivity analysis could be conducted due to the limited number of studies and all of them having methodological insufficiencies.

All analyses were run in Stata version 14.0 (StataCorp LP) by one author (SNP), and the data set was openly provided^24^ with a 5% significance level.

## 3 RESULTS

### 3.1 Study selection

**FIGURE 1:**
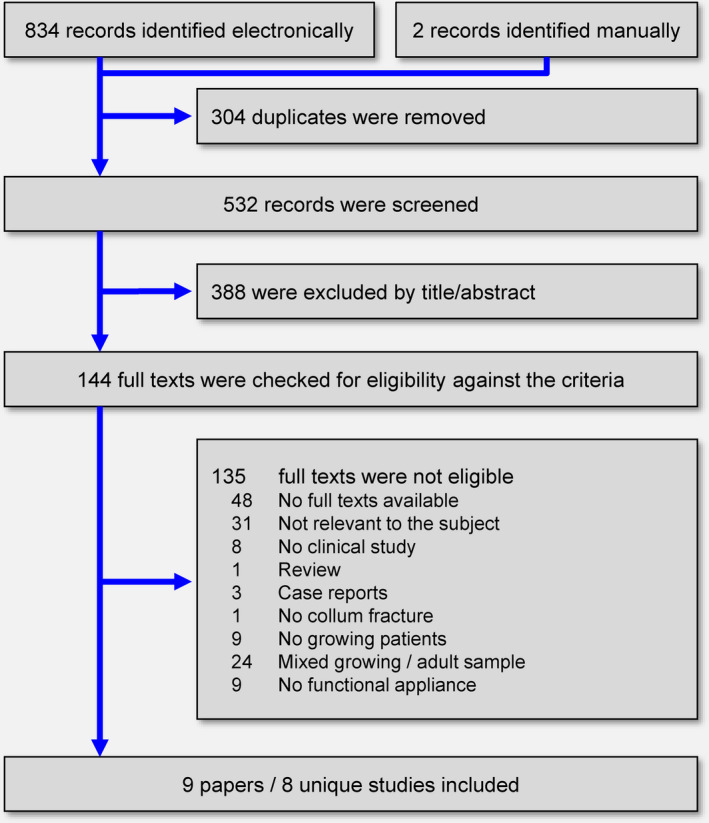
Flow diagram for the identification and selection of eligible studies

The electronic literature search yielded 834 results, while another two were manually identified from the reference/citation lists of identified papers (Figure 1). After duplicate removal and screening the titles/abstracts of identified reports, the full texts of 144 papers were checked against the eligibility criteria (Supplement 3). Ultimately, 9 papers pertaining to 8 unique studies (1 prospective and 7 retrospective non‐randomised) were included, which were published as journal papers.

### 3.2 Study characteristics

The included studies were conducted in university clinics in Austria, China and Germany (Table 1). A total of 223 patients treated with functional appliances were included, with a median sample of 26 patients per included study (range 7 to 44 patients per study). Out of the four studies reported on patient sex, 78 of the 141 patients in total were male (55%), while the patients' age ranged from 3 to 16 years.

**TABLE 1:** Characteristics of included studies

Study	Design; setting; country^a^	Patients (M/F); age^b^	Unilateral/bilateral	Categorisation (S&S)	Treatment	Follow‐up in years
Kahl 1990	rNRS; Uni; DEU	G1–3: 21 (NR); (3.9–13.7)	G1–3: 18/3	I 0%; II 0%; III 52%; IV 19%; V 29%; VI 0%	G1: Activator G2: IMF & Activator G3: IMF	NR (1.3–4.0)
Kahl 1995	rNRS; Uni; DEU	19 (NR); (3.0–9.0)	17/2	Ι 38%; ΙΙ 14%; ΙΙΙ 5%; ΙV 10%; V 33%; VI 0%	Activator	ca.5.4 (3.0–9.6)
Kahl‐Nieke 1994	rNRS; Uni; DEU	7 (4/3); (4.0–9.0)	7/0	I 14%; II 29%; III 14%; IV 14%; V 29%; VI 0%	Activator	6.3 (5.0–10.0)
Kahl‐Nieke 1995	rNRS; Uni; DEU	12 (NR); (5.0–16.0)	8/4	I 19%; II 0%; III 19%; IV 31%; V 31%; VI 0%	Activator	1.5 (1.5)
Kahl‐Nieke 1998; 1999	rNRS; Uni; DEU	G1: 19 (10/9); 9.0 G2: 20 (10/10); 8.4	G1: 19/0 G2: 20/0	I 31%; II 10%; III 10%; IV 13%; V 36%; VI 0%	Activator	G1/2: 4.9–5.0 (NR)
Liu 2014	rNRSl Uni; CHN	30 (18/12); (4.0–8.0)	23/7	NR	Occlusal splint	3.5 (1.0–6.0)
Strobl 1999	pNRS; Uni; AUT	55 (37/18); (2.5–9.8)	55/0	I 11%; II 13; III 42%; IV 13%; V 18; VI 4%	Activator	“through the period of growth”
Zhao 2014	rNRS; Uni; CHN	40 (17/23); (6.0–16.0)	27/13	I 0%; II 5%; III 23%; IV 10%; V 43%; VI 20%	Occlusal splint	NR (1.2–4.0)

Abbreviations: G, group; IMF, intermaxillary fixation; M/F, male/female; NR, not reported; pNRS, prospective non‐randomised study; retrospective non‐randomized study; rNRS; S&S, Spiessl & Schroll categorisation; Uni, university clinic.

^a^ Given as 3‐letter ISO coding.

^b^ Given in years as range (2 values in parenthesis) or mean (one value).

In three of the included studies, only unilateral condyle fractures were included, while the remaining five studies included both unilateral and bilateral fractures. Seven of the included studies used the categorisation of condylar fractures proposed by Spiessl and Schroll.^15^ According to this categorisation, from the 194 assessed condylar fractures 15% (*n* = 29) were condylar fractures without angulation and dislocation (Type I), 9% (*n* = 18) were low condylar fractures with angulation (Type II), 26% (*n* = 51) were high condylar fractures with angulation (Type III), 14% (*n* = 27) were low condylar fractures with dislocation (Type IV), 31% (*n* = 59) were high condylar fractures with dislocation (Type V), and 5% (*n* = 10) were fractures of the condylar head (Type VI).

Orthopaedic treatment was done with activator appliances in six of the studies and with occlusal splints in the remaining two studies. One study (Kahl and Gerlach, 1990)^8^ also compared three different groups: one with only functional appliance treatment, one with intermaxillary fixation and then functional appliance treatment, and one with intermaxillary fixation and functional exercises. Follow‐up after condylar fracture ranged from 1 to 10 years, and patients were evaluated clinically or radiographically.

### 3.3 Risk of bias within studies

The included non‐randomised studies presented several issues that increased their risk for bias (Table 2). Seven of the 8 studies were retrospective, while the patient's sex and skeletal maturation age were often inadequately described. In half of the included studies (50%), selection bias could exist, as patient selection was based on factors that could be associated with the outcome of treatment. No study blinded the outcome assessor, and only 3 studies (38%) included a relatively adequate sample with at least 25 patients. Follow‐up periods were relatively adequate, and only one study (13%) did not have a minimum follow‐up of a year. From the two studies that made comparisons between groups (Kahl and Gerlach, 1990; Kahl‐Nieke and Fischbach, 1998),^8^, ^25^ matching according to patient characteristics was judged to be adequate only for one of them, while the observation period was not common across groups for both of the studies.

**TABLE 2:** Methodological characteristics of included studies

Question	Kahl 1990	Kahl 1995	Kahl‐Nieke 1994	Kahl‐Nieke 1995	Kahl‐Nieke 1998; 1999	Liu 2014	Strobl 1999	Zhao 2014
Was the study prospective?	No	No	No	No	No	No	Yes	No
Were participants age described?	Yes	Yes	Yes	Yes	Yes	Yes	Yes	Yes
Were participants sex described?	No	No	Yes	No	Yes	Yes	Yes	Yes
Were participants maturation stage described?	No	No	No	No	No	No	No	No
Was the fracture type adequately described?	Yes	Yes	Yes	Yes	Yes	No	Yes	Yes
Was FA (functional appliance) treatment described?	Yes	Yes	Yes	Yes	Yes	Yes	Yes	Yes
Was selection of patients based on any factor that could influence the outcome (fracture type, malocclusion, compliance, missed appointments, breakages, attrition)?	Unclear	Yes	Yes	Unclear	Probably yes	Unclear	Yes	Unclear
Were outcomes patients measured blindly?	No	No	No	No	No	No	No	No
Was the adequate sample? (25 patients per group)	No	No	No	No	No	Yes	Yes	Yes
Was there adequate follow‐up (at least 1 year)?	Yes	Yes	Yes	No	Yes	Yes	Yes	Yes
Were FA/ CTR groups clearly defined?	Yes	—	—	—	Yes	—	—	—
Were FA/ CTR patients treated/observed at the same place/time?	Unclear	—	—	—	Unclear	—	—	—
Were FA/ CTR patients matched for baseline age?	No	—	—	—	Yes	—	—	—
Were FA/ CTR patients matched for baseline sex?	NR	—	—	—	Yes	—	—	—
Were FA/ CTR patients matched for baseline malocclusion?	NR	—	—	—	NR	—	—	—
Were FA/ CTR patients matched for fracture type?	No	—	—	—	Unclear	—	—	—
Was the use of other treatments the same among FA/ CTR patients?	Yes	—	—	—	Yes	—	—	—
Was the observation period similar for FA/ CTR patients?	No	—	—	—	No	—	—	—
Were FA/CTR patients measured exactly the same way?	Yes	—	—	—	Yes	—	—	—

Note

CTR Control group.

Abbreviations: FA functional appliance; NR not reported.

### 3.4 Results of individual studies and data synthesis

The included studies reported on a wide variety of outcomes after functional appliance treatment. Kahl et al reported that 3.0–9.6 years after the fracture and the subsequent functional appliance treatment 42%–58% of the patients had returned to good or normal function, while 14%–28% reported noises or pain on palpation. Computerised tomography (CT) indicated altered condyle shape in 67% of the fractured condyles, bony spurs in 24% of the condyles and neo‐arthrosis in 10% of the condyles. Often were also seen deviations in the condylar axis, in the length of the condylar neck, in the curvature of the articular eminence and the width of the joint space. Similar findings of alterations of size, shape, bony remodelling and position of the condyle on the fracture side, as well as adaptive changes in the temporal component of the joint, were reported by Kahl‐Nieke et al.^26^ Another study^27^ indicated that bone remodelling might be associated with the severity of the condylar fracture, as fractures of types IV and V according to the Spiessl and Schroll categorisation (condylar fractures with dislocation) were more often remodelled more badly than less severe fractures. Strobl et al^12^ found that after 1 year of activator treatment following collum fracture, bone remodelling was complete in all cases with adaptive changes. Younger patients (2–6 years old) showed considerably better outcomes than older patients (7–10 years old), who occasionally had incomplete regeneration, condylar deformity, shorter condylar heads or hypertrophic condylar deformity. However, in all instances no joint ankylosis, and no disturbance of mandibular or facial growth was detected throughout the patients' growth period.

Two studies^10^, ^28^ reported on orthopaedic treatment with occlusal splints instead of conventional functional appliances like the activator. Liu et al^10^ found excellent or good clinical outcomes for all patients with full radiological remodelling seen in 63% and partial remodelling in the remaining 37% of the patients after 1 year. CT analysis indicated slightly larger maximum anteroposterior and mediolateral diameters of the fractured condyles compared with the healthy condyles, but no patient had any complications. Zhao et al^28^ found that no patient showed 1.2–4.0 years after the fracture ankylosis, malocclusion, functional disturbance or facial asymmetry. No titling of the occlusal plane for unilateral fractures and no open bite for bilateral fractures was seen. After 1 year, the majority of assessed patients (38 out of 40) had midline deviation during mouth opening less than 3 mm and all exhibited good protrusive and lateral movements.

All included studies reported aggregate data in the paper, while for one study^29^ data were extracted from boxplots. Two studies^8^, ^25^ also provided individual patient data in tables, which were extracted and re‐analysed.

The study of Kahl and Gerlach^8^ compared three different groups (Supplement 4). No significant differences were found between functional appliance treatment versus intermaxillary fixation and then functional appliance treatment regarding mouth opening (*p* = .27), midline deviation (*p* = .73) or joint auscultation findings (*p* = .32). Similarly, no differences were found between functional appliance treatment versus intramaxillary fixation and then functional exercises regarding mouth opening (*p* = .68), midline deviation (*p* = .83) or joint auscultation (*p* = .21).

The study of Kahl‐Nieke and Fischbach^25^, ^30^ compared among patients treated with functional appliances the dimensions/characteristics of the injured condyles with the contralateral healthy ones (Supplement 5–6). Fracture categorisation was shown to be associated with treatment outcome, as in Type IV fractures (low condylar fracture with dislocation) the injured condyle had greater mesiodistal size difference to the contralateral condyle compared with Type I fractures (condylar fracture without angulation and dislocation) (difference = 0.31 mm; 95% CI = 0.02 to 0.59 mm; *p* = .04). Additionally, better outcome was seen for boys patients, as they had smaller difference than girls in the mesiodistal size of the injured condyle compared with the contralateral healthy condyle (difference = −0.21 mm; −0.36 to −0.07; *p* = .006). When taking a ±10% difference as a cut‐off, male patients had significantly more often injured/healthy condyles of similar mesiodistal size (OR = 31.5; 95% CI = 2.35 to 422.30; *p* = .009) than female patients. At the same time, condyle fractures with dislocation were associated with considerably worse outcome in terms of the injured/healthy condyles having roughly the same mesiodistal size (OR = 0.07; 95% CI = 0.01 to 0.68; *p* = .02). No other significant effects of fracture type, patient age, patient sex or follow‐up duration were seen for anteroposterior condyle dimensions (Supplement 5), condylar volume (Supplement 6) or condylar bone density (Supplement 6).

**FIGURE 2:**
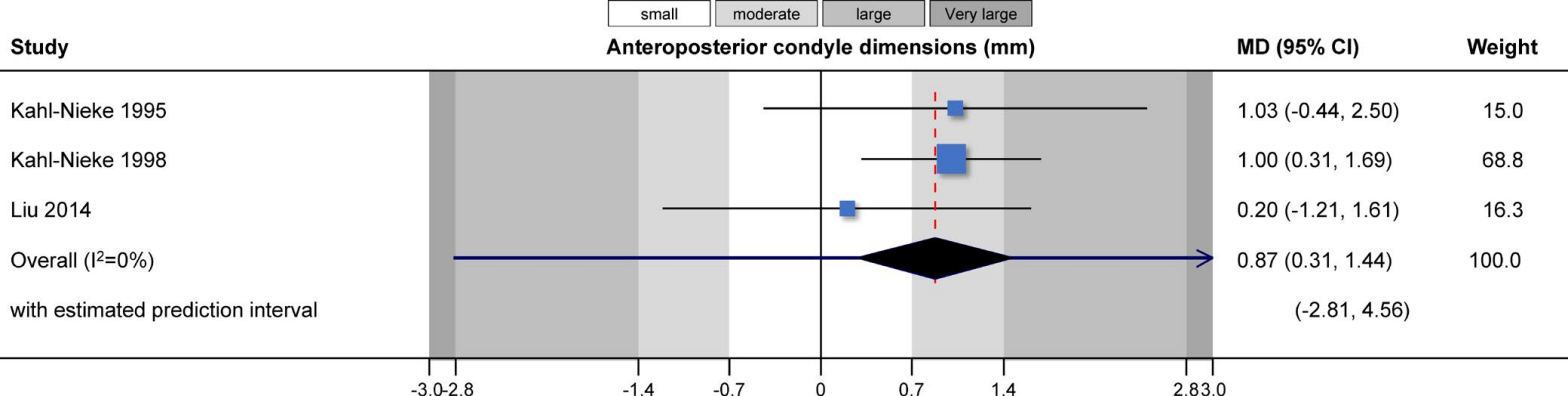
Contour‐enhanced forest plot for the comparison of anteroposterior condyle dimensions between treated and untreated groups. CI, confidence interval; MD, mean difference

**FIGURE 3:**
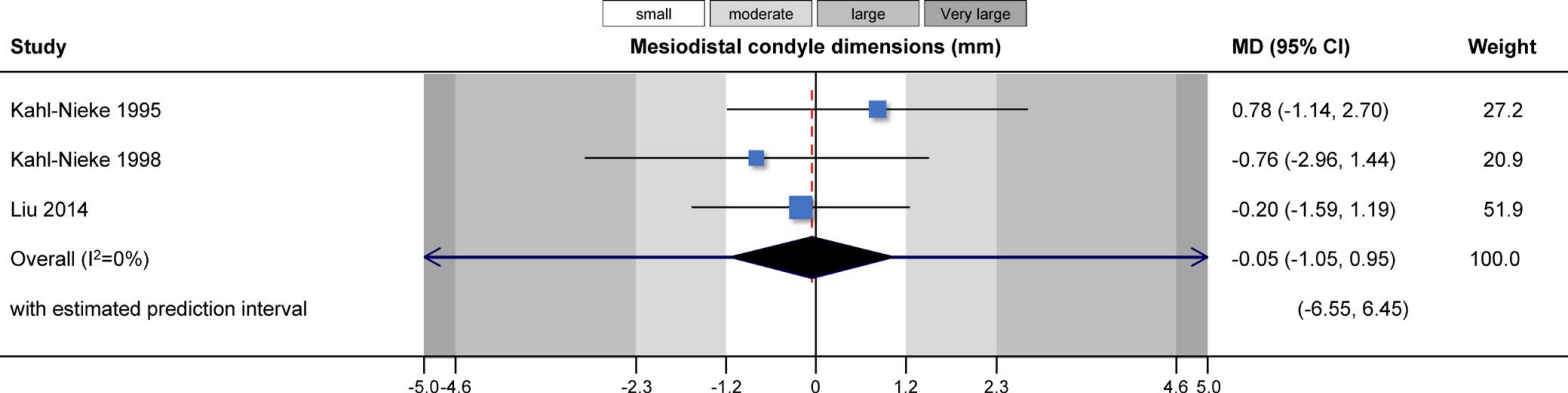
Contour‐enhanced forest plot for the comparison of mesiodistal condyle dimensions between treated and untreated groups. CI, confidence interval; MD, mean difference

Direct comparisons within and across studies could be made in only a handful of instances (Table 3). Meta‐analysis of three studies^10^, ^25^, ^27^ indicated that functional appliance treatment was associated with greater anteroposterior condyle dimensions of the injured condyle compared with the contralateral healthy condyle (MD = 0.87 mm; 95% CI = 0.30 to 1.45 mm; *p* = .003; Figure 2). Another meta‐analysis of three studies^10^, ^25^, ^27^ found no significant difference in the mesiodistal condyle size between the injured and the contralateral healthy joint (MD = −0.05 mm; 95% CI = −1.05 to 0.95 mm; *p* = .92; Figure 3). A single study^27^ found that the collum length was significantly smaller at the injured joint side compared with the contralateral healthy side (MD = −2.89 mm; 95% CI =− 5.29 to −0.49 mm; *p* = .02), and a tendency for a smaller condylar angle (MD = −7.86°; 95% CI = −16.07 to 0.35°; *p* = .06).

**TABLE 3:** Meta‐analytical comparison of the injured condyle to uninjured condyles

Outcome	Studies	MD (95% CI)	*p*	I^2^ (95% CI)	τ^2^ (95% CI)	95% prediction
Anteroposterior condyle dimensions (mm)	3	0.87 (0.30, 1.45)	.003	0% (0%, 50%)	0 (0, 3.71)	−2.85, 4.59
Mesiodistal condyle dimensions (mm)	3	−0.05 (−1.05, 0.95)	.92	0% (0%, 92%)	0 (0, 9.16)	−6.54, 6.45

Abbreviations: CI, confidence interval; HU, Hounsfield units; MD, mean difference.

### 3.5 Additional analyses, risk of bias across studies, sensitivity analyses and quality of evidence

Several subgroup analyses, meta‐regressions, assessments for reporting biases and sensitivity analyses were originally planned in the review's protocol but could ultimately not be performed due to limited data and inadequate reporting (Supplement 1).

The quality of evidence according to GRADE was very low for all meta‐analyses, since only non‐randomised and especially retrospective clinical studies with many methodological issues were available. This means that further research in terms of well‐designed studies is very likely to have an important impact, which is likely to change our current estimates of effect.

## 4 DISCUSSION

### 4.1 Summary of evidence

The current systematic review summarises evidence from non‐randomised clinical studies on mandibular condyle fracture treatment outcome with functional appliances. Out of the initially identified 834 hits from the literature search, 8 studies (involving 223 patients) were ultimately included.

The identified studies indicated that the functional appliances might lead to good clinical rehabilitation of fractured mandibular condyles in growing patients. The rationale behind this approach is that early controlled mobilisation of the mandible using a functional appliance results in a re‐instatement of an organised functional condyle,^6^ while late complications such as ankylosis, facial growth disturbances or functional joint disorders are prevented.^31^

Meta‐analysis of three studies indicated that after functional appliance treatment the condyle of the affected size was significantly larger on the anteroposterior dimension than the condyle of the non‐affected side (MD = 0.87 mm; *p* = .003). This might indicate that the anterior repositioning of the mandible acts as a stimulant that ultimately lead to bone remodelling in the condylar area. This was shown decades ago to be feasible in animal studies^32^, ^33^ and was confirmed by subsequent human studies on Class II malocclusion.^11^ However, no correlation has been found between an increased size of the condylar head and the functional status of the stomatognathic system.^34^ This new bone growth is associated at the same time with an 10%–70% decrease in muscle volume^30^ and an overcompensation of function from the contralateral healthy side, where a 20%–40% increase in the volume of the lateral pterygoid muscle can be seen.

The prognosis of the condylar rehabilitation seems to be mainly affected by the type of the mandibular fracture—namely its localisation and the existence of dislocation. Low fracture types and fractures with dislocation might be associated more with shortening of the condylar process and excessive bone overgrowth that high fractures and fractures without dislocation.^25^ Re‐analysis of that study's data (Supplement 5) indicated that patients with luxated fractures had significantly smaller odds of having a condylar head of size (±10%) to the healthy unaffected condyle compared with patients without luxation. Similar results have been given by reports of cases,^34^ where high fractures showed a high degree of remodelling, while some presented a notching of the lateral surface of the condylar head and a slight medial deviation of the condylar head. Low fractures with luxation on the other side presented often unfavourable remodelling, with irregular condylar morphology and altered topography related to the glenoid fossa, but still with muscular adaptation and no functional disturbances. However, especially in patients with low condylar fractures, residual facial asymmetries and malocclusions can be seen,^35^ which might even need to be corrected with orthognathic surgery.

Remodelling prognosis of the fractured condyle seems also to be closely associated with the age of the patient.^25^ Previous studies indicate that in patients up to 10 years of age at the time of the mandibular fracture, the development of a new condylar head might be more probable than older patients.^2^, ^3^, ^36^ It is, however, important to note that even though younger patients have better chances for an initial remodelling of the condyle, achievement of normal condylar morphology may require several years,^5^ and therefore, long follow‐up of these children is warranted.

In the included studies, only two different functional appliances were used (either activator or occlusal splint). It has been previously reported that considerable variation exists in both the skeletal and dental effects among the various removable functional appliances, with more stable appliances with clasps such as the Twin Block having a potential advantage.^37^ On the other hand, loose fitting appliances such as the activator/monobloc might encourage activation of the protractor and elevator muscles to keep it in place^38^ and be preferable in the orthopaedic rehabilitation of condylar fractures. It might be possible that different appliance designs have different effectiveness during rehabilitation of fractured condyles, but existing evidence is very limited to enable such direct comparisons.

### 4.2 Strengths and limitations

This systematic review has several strengths, comprising an a priori registered protocol,^39^ a comprehensive literature search, the inclusion of randomised or matched non‐randomised studies, the use of modern analytic methods,^19^ the application of the GRADE approach to assess the strength of provided recommendations^22^ and the transparent provision of all data.^40^

Some limitations also do exist in the present review. For one, methodological issues existed for all included studies that might influence conclusions, and this is especially the case for included retrospective non‐randomised studies.^41^, ^42^ Furthermore, all meta‐analyses were based predominantly on small trials, which might affect the precision of the estimates.^43^ Additionally, the small number of studies with limited samples that were ultimately included in the meta‐analyses and their incomplete reporting of results and potential confounders precluded the conduct of many analyses for subgroups and meta‐regressions that might enable identification of patient subgroups for which functional appliances might be most effective.

## 5 CONCLUSIONS

There is currently very limited evidence on the treatment of growing patients with mandibular fractures using orthopaedic functional appliances. Some data indicate that functional appliance treatment is associated with partial or complete remodelling of the fractured condyle and the temporomandibular joint with acceptable clinical results. However, existing studies are mostly old, single‐group cohort studies without control groups and with many methodological issues and uncertainty still exist around the long‐term outcomes of functional appliances for condylar fractures.

## CONFLICT OF INTEREST

None to declare.

### PEER REVIEW

The peer review history for this article is available at https://publons.com/publon/10.1111/joor.13178.

## Supporting information

Supplementary Material

## Data Availability

The full data set is openly provided through Zenodo (http://doi.org/10.5281/zenodo.4553859).
