# Supplementary material for: Functional appliance treatment for mandibular fractures: A systematic review with meta‐analyses
Source: J Oral Rehabil. 2021 May 24;48(8):945–54. doi: 10.1111/joor.13178 (PMC8362118; doi:10.1111/joor.13178)
Supplement: Supplementary file 1 — Supplementary Material [file JOOR-48-945-s001.docx]

**Functional appliance treatment for mandibular fractures: a systematic review**

**Supplementary material**

**Supplement 1**. Additional details on the used methods and deviations from protocol.

- Quantitative data were extracted from an included study providing box plots using the online program WebPlotDigitizer (https://automeris.io/WebPlotDigitizer/).
- The individual patient data available from the included studies were re-analyzed with linear regression (reporting unstandardized coefficients) or logistic regression (reporting odds ratios). After the initial crude models, additional adjusted models were constructed with patient age, sex, and fracture categorization as covariates.
- Initially, possible sources of heterogeneity in meta-analyses were to be sought through pre-specified mixed-effects subgroup analyses and random-effects meta-regression in cases of at least five meta-analyzed studies. Pre-defined subgroup analyses included: (i) subsets according to the patient sample characteristics (age, sex); (ii) subsets according to the treatment characteristics (conservative treatment, surgical treatment including functional appliance therapy, type of functional appliance administered); (iii) subsets according to the exact fracture location (according to the classification of fractures of the head and neck of the condyle according to Spiessl and Schroll). These could not ultimately be conducted as only 2 meta-analyses with 3 studies each were included.
- Similarly, sensitivity analyses had been planned, but no high quality studies were identified.
- Finally, reporting biases could not be assessed, as less than 10 studies were included in all meta-analyses.

**Supplement 2**. Literature searches performed (last searchdate: October 1, 2020) with the corresponding hits.

| **Nr** | **Database** | **Search** | **Limits** | **Hits** |
| --- | --- | --- | --- | --- |
| 1 | MEDLINE (via PubMed) | (mandib* OR jaw*) AND (condyl* OR collum* OR intracapsular* OR extracapsular* OR decapitular*) AND (fractur* OR break*) AND (orthodon* OR orthoped* OR orthopaed* OR "functional appliance" OR "bite jumping" OR "anterior repositioning" OR "mandibular advancement" OR Activator OR Andresen OR Bass OR Bimler OR Biobloc OR Bionator OR Frankel OR Fränkel OR Fraenkel OR Harvold OR Herbst OR Monobloc OR Stockli OR Stoeckli OR Stöckli OR Teuscher OR "Twin Block" OR Twinblock) | Human | 255 |
| 2 | Embase | Same as MEDLINE | Human | 53 |
| 3 | CDSR | Same as MEDLINE | - | 0 |
| 4 | DARE | Same as MEDLINE | - | 0 |
| 5 | CENTRAL | Same as MEDLINE | - | 6 |
| 6 | Scopus | ( TITLE-ABS-KEY ( ( mandib* OR jaw* ) ) AND TITLE-ABS-KEY ( ( condyl* OR collum* OR intracapsular* OR extracapsular* OR decapitular* ) ) AND TITLE-ABS-KEY ( ( fractur* OR break* ) ) AND TITLE-ABS-KEY ( ( orthodon* OR orthoped* OR orthopaed* OR "functional appliance" OR "bite jumping" OR "anterior repositioning" OR "mandibular advancement" OR activator OR andresen OR bass OR bimler OR biobloc OR bionator OR frankel OR fränkel OR fraenkel OR harvold OR Herbst OR Monobloc OR Stockli OR Stoeckli OR Stöckli OR Teuscher OR "Twin Block" OR Twinblock) ) ) | Dentistry | 158 |
| 7 | Web of Knowledge | Same as MEDLINE | DENTISTRY ORAL SURGERY MEDICINE | 326 |
| 8 | Virtual Health Library | Same as MEDLINE | - | 36 |

CDSR, Cochrane Database of Systematic Reviews; CENTRAL, Cochrane Central Register of Controlled Trials; DARE, Database of Abstracts of Reviews of Effects.

**Supplement 3**. List of studies identified from the literature search with inclusion / exclusion status.

| **Nr** | **Paper** | Status |
| --- | --- | --- |
| 1 | Aas AL, Skaare AB. Management of a 9-year-old boy experiencing severe dental injury--a 21-year follow-up of three autotransplants: a case report. Dent Traumatol. 2011;27(6):468-72. | Excluded by title |
| 2 | Abdel-Galil K, Loukota R. Fixation of comminuted diacapitular fractures of the mandibular condyle with ultrasound-activated resorbable pins. Br J Oral Maxillofac Surg. 2008;46(6):482-4. | Excluded by title |
| 3 | Agrawal R, Patel DP, Desai BB. Correction of severe facial asymmetry: A case with fractured condyle. Apos Trends in Orthodontics. 2019;9(1):59-64. | Excluded by title |
| 4 | Albogha MH, Mori Y, Takahashi I. Three-dimensional titanium miniplates for fixation of subcondylar mandibular fractures: Comparison of five designs using patient-specific finite element analysis. J Craniomaxillofac Surg. 2018;46(3):391-7. | Excluded by title |
| 5 | Al-Moraissi EA, Louvrier A, Colletti G, Wolford LM, Biglioli F, Ragaey M, et al. Does the surgical approach for treating mandibular condylar fractures affect the rate of seventh cranial nerve injuries? A systematic review and meta-analysis based on a new classification for surgical approaches. J Craniomaxillofac Surg. 2018;46(3):398-412. | Excluded by title |
| 6 | Amaral MB, Bueno SC, Silva AA, Mesquita RA. Superolateral dislocation of the intact mandibular condyle associated with panfacial fracture: a case report and literature review. Dent Traumatol. 2011;27(3):235-40. | Excluded by title |
| 7 | Anehosur V, Kulkarni K, Shetty S, Kumar N. Clinical outcomes of endoscopic vs retromandibular approach for the treatment of condylar fractures-a randomized clinical trial. Oral Surgery Oral Medicine Oral Pathology Oral Radiology. 2019;128(5):479-84. | Excluded by title |
| 8 | Anonymous. 31st Annual Scientific Conference and Exhibition of the American-College-of-Oral-and-Maxillofacial-Surgeons/Concurrent Preconference Scientific Sessions, San Antonio, TX, USA, April 25 -27, 2010. Oral Surgery Oral Medicine Oral Pathology Oral Radiology and Endodontology. 2010;110(3):310-8. | Excluded by title |
| 9 | Aoshima O, Nakamura T, Satoh Y, Nakano Y, Nagano H, Imamura R, et al. Orthodontic treatment of an adult male after bilateral condylectomy of the mandible for injuries sustained in a traffic accident. J Oral Sci. 1998;40(1):1-8. | Excluded by title |
| 10 | Aquilina P, Parr WCH, Chamoli U, Wroe S, Clausen P. A biomechanical comparison of three 15-mm plate and screw configurations and a single 2.0-mm plate for internal fixation of a mandibular condylar fracture. Craniomaxillofacial Trauma and Reconstruction. 2014;7(3):218-23. | Excluded by title |
| 11 | Aquilina P, Parr WCH, Chamoli U, Wroe S. Finite element analysis of patient-specific condyle fracture plates: A preliminary study. Craniomaxillofacial Trauma and Reconstruction. 2015;8(2):111-6. | Excluded by title |
| 12 | Aronovich S. Skeletal reconstruction of craniofacial deformities with alloplastic total joint components and orthognathic surgery. Cleft Palate-Craniofacial Journal. 2016;53(4):e101-e2. | Excluded by title |
| 13 | Asprino L, Consani S, De Moraes M. A comparative biomechanical evaluation of mandibular condyle fracture plating techniques. Journal of Oral and Maxillofacial Surgery. 2006;64(3):452-6. | Excluded by title |
| 14 | Baker B, Gibbons S, Woods M. Intra-alveolar distraction osteogenesis in preparation for dental implant placement combined with orthodontic/orthognathic surgical treatment: a case report. Aust Dent J. 2003;48(1):65-8. | Excluded by title |
| 15 | Baraglia M. The use of metal clips in Maxillo-Facial surgery. Rivista Italiana di Chirurgia Plastica. 1995;27(2):237-9. | Excluded by title |
| 16 | Barker TA. Facial Injury - Football. Medicine and Science in Sports and Exercise. 2001;33(5):S309-S. | Excluded by title |
| 17 | Bavia PF, Keith DA. Isolated tympanic plate fracture and associated temporomandibular joint symptoms Literature review and report of 2 cases. Journal of the American Dental Association. 2019;150(9):794-8. | Excluded by title |
| 18 | Becktor JP, Rebellato J, Sollenius O, Vedtofte P, Isaksson S. Transverse Displacement of the proximal segment after bilateral sagittal osteotomy: A comparison of lag screw fixation versus miniplates with monocortical screw technique. Journal of Oral and Maxillofacial Surgery. 2008;66(1):104-11. | Excluded by title |
| 19 | Belli E, Matteini C, Incisivo V. Orthodontic-surgical treatment after posttraumatic bilateral condylectomy of the mandible in an adult patient. J Craniofac Surg. 2003;14(1):55-62. | Excluded by title |
| 20 | Ben-Bassat Y, Brin I, Jarjoura R, Regev E. Morphological occlusal features following condylar fractures in children. Eur J Orthod. 2012;34(2):147-51. | Excluded by title |
| 21 | Biglioli F, Colletti G. Transmasseter approach to condylar fractures by mini-retromandibular access. J Oral Maxillofac Surg. 2009;67(11):2418-24. | Excluded by title |
| 22 | Birke WP. [Use of percutaneous screws in the middle face]. Dtsch Stomatol. 1973;23(5):344-7. | Excluded by title |
| 23 | Boccaccio A, Cozzani M, Pappalettere C. Analysis of the performance of different orthodontic devices for mandibular symphyseal distraction osteogenesis. Eur J Orthod. 2011;33(2):113-20. | Excluded by title |
| 24 | Boffano P, Roccia F, Gallesio C, Karagozoglu KH, Forouzanfar T. Bicycle-related maxillofacial injuries: a double-center study. Oral Surgery Oral Medicine Oral Pathology Oral Radiology. 2013;116(3):275-80. | Excluded by title |
| 25 | Borges NC, Weissengruber GE, Huber J, Kofler J. Ultrasonographic imaging of the temporomandibular joint in healthy cattle and pathological findings in one clinical case. New Zealand Veterinary Journal. 2016;64(6):330-6. | Excluded by title |
| 26 | Bottini DJ, Gnoni G, Grimaldi M, Cervelli G, Cervelli V. [Skull base fractures with TMJ involvement: literary review and case report]. Ann Ital Chir. 2003;74(1):85-90; discussion 1. | Excluded by title |
| 27 | Cadenat H, Boutault F, Comiti G. Interest of surgical treatment of subcondylar fractures. Revue de Stomatologie et de Chirurgie Maxillo-Faciale. 1984;85(6):455-64. | Excluded by title |
| 28 | Ceccarelli AA, Rivas NH, Lorenz GI. Recuperación morfofuncional de la ATM postcondilectomía, tratada con ortopedia funcional de los maxilares. Rev Asoc Argent Ortop Funcional Maxilares. 2001;32(1):27-36. | Excluded by title |
| 29 | Champy M, Loddé JP, Schmitt R, Jaeger JH, Muster D. Mandibular osteosynthesis by miniature screwed plates via a buccal approach. J Maxillofac Surg. 1978;6(1):14-21. | Excluded by title |
| 30 | Chang L-R, Chen C-C, Jeng SF, Chen Y-R, Hwang L-C, Lin T-S. Investigation of a Modified Novel Technique in Bilateral Sagittal Splitting Osteotomy Fixation: Finite Element Analysis and In Vitro Biomechanical Test. Biomed Research International. 2020;2020. | Excluded by title |
| 31 | Chen CY, Chang LR, Chen WH, Lin LW. Reduction of mandible fractures with direct bonding technique and orthodontic appliances: two case reports. Dent Traumatol. 2010;26(2):204-9. | Excluded by title |
| 32 | Choi BH, Yoo JK. Open reduction of condylar neck fractures with exposure of the facial nerve. Oral Surgery Oral Medicine Oral Pathology Oral Radiology and Endodontology. 1999;88(3):292-6. | Excluded by title |
| 33 | Christou T, Kau CH, Abou-Kheir NS, Louis PJ. The use of three-dimensional evaluation in the management of a complex patient with mandibular fracture: a 5-year evaluation. J Craniofac Surg. 2014;25(3):e223-8. | Excluded by title |
| 34 | Chul SH, 김지연. Prophylactic Surgical Extractions of Impacted Third Molars in Young Patients : Journal Review. Journal of Korean academy of Advanced General Dentistry. 2018;7(1):23-9. | Excluded by title |
| 35 | Costa FWG, Bezerra MF, Ribeiro TR, Pouchain EC, Sabóia VdPA, Soares ECS. Biomechanical analysis of titanium plate systems in mandibular condyle fractures: a systematized literature review. Acta cir bras. 2012;27(6):424-9. | Excluded by title |
| 36 | Cucurullo R, Giannuzzi I, Clivio A, Biagi R. Management of unilateral condylar fracture in a 9.6-year-old female. Eur J Paediatr Dent. 2009;10(2):95-101. | Excluded by title |
| 37 | de Sales MA, do Amaral JI, de Amorim RF, de Almeida Freitas R. Bifid mandibular condyle: case report and etiological considerations. J Can Dent Assoc. 2004;70(3):158-62. | Excluded by title |
| 38 | Defabianis P. Treatment of condylar fractures in children and youths: the clinical value of the occlusal plane orientation and correlation with facial development (case reports). J Clin Pediatr Dent. 2002;26(3):243-50. | Excluded by title |
| 39 | Donoff RB, Roser SM. Management of condylar fractures in patients with cervical spine injury: report of cases. J Oral Surg. 1973;31(2):130-5. | Excluded by title |
| 40 | Farronato G, Giannini L, Galbiati G, Maspero C. Long term results of open reduction management of condylar fracture: a 20 years follow-up. Case report. Minerva Stomatol. 2012;61(10):457-65. | Excluded by title |
| 41 | Fumagalli G, Piazzini E, Zampetti G. [Comments on the treatment of fractures of the mandibular condyle in young patients]. Dent Cadmos. 1967;35(9):1253-61. | Excluded by title |
| 42 | Girthofer K, Göz G. TMJ remodeling after condylar fracture and functional jaw orthopedics a case report. J Orofac Orthop. 2002;63(5):429-34. | Excluded by title |
| 43 | Harstall R, Gratz KW, Zwahlen RA. Mandibular condyle dislocation into the middle cranial fossa: a case report and review of literature. J Trauma. 2005;59(6):1495-503. | Excluded by title |
| 44 | Heggie AA. Concepts in the management of temporomandibular ankylosis. Ann R Australas Coll Dent Surg. 1996;13:132-5. | Excluded by title |
| 45 | Johal AS, Davies SJ, Franklin CD. Condylar metastasis: a review and case report. Br J Oral Maxillofac Surg. 1994;32(3):180-2. | Excluded by title |
| 46 | Kober C, Stübinger S, Hellmich C, Sader R, Zeilhofer HF. Mandibular finite element simulation as a tool for trauma surgery. Int J Comput Dent. 2008;11(3-4):175-81. | Excluded by title |
| 47 | Kragstrup TW, Christensen J, Fejerskov K, Wenzel A. Frey syndrome-an underreported complication to closed treatment of mandibular condyle fracture? Case report and literature review. J Oral Maxillofac Surg. 2011;69(8):2211-6. | Excluded by title |
| 48 | Lejman T, Sułko J. [Orthopedic problems in children with congenital insensitivity to pain]. Chir Narzadow Ruchu Ortop Pol. 1999;64(2):169-75. | Excluded by title |
| 49 | Ma J, Ma L, Wang Z, Zhu X, Wang W. The use of 3D-printed titanium mesh tray in treating complex comminuted mandibular fractures: A case report. Medicine (Baltimore). 2017;96(27):e7250. | Excluded by title |
| 50 | Miller JR. Confounding. Am J Orthod Dentofacial Orthop. 2014;145(5):706-8. | Excluded by title |
| 51 | Nishio C, Tanimoto K, Hirose M, Horiuchi S, Kuroda S, Tanne K, et al. Stress analysis in the mandibular condyle during prolonged clenching: a theoretical approach with the finite element method. Proc Inst Mech Eng H. 2009;223(6):739-48. | Excluded by title |
| 52 | Paphangkorakit J, Osborn JW. Proclination of lower incisors: a design to maximize food penetration and minimize torque. J Oral Rehabil. 2008;35(11):870-4. | Excluded by title |
| 53 | Park JH, Tai K, Sato Y. Orthodontic treatment of a patient with severe crowding and unilateral fracture of the mandibular condyle. Am J Orthod Dentofacial Orthop. 2016;149(6):899-911. | Excluded by title |
| 54 | Pini CE, Merlini C. [Traumatic lesions of the temporomandibular articulation]. Minerva Stomatol. 1969;18(11):644-64. | Excluded by title |
| 55 | Pistolas P. What would you do? Int J Orthod Milwaukee. 2009;20(3):27-32. | Excluded by title |
| 56 | Puelacher WC, Waldhart E. Miniplate eminoplasty: a new surgical treatment for TMJ-dislocation. J Craniomaxillofac Surg. 1993;21(4):176-8. | Excluded by title |
| 57 | Shen G. The role of type X collagen in facilitating and regulating endochondral ossification of articular cartilage. Orthod Craniofac Res. 2005;8(1):11-7. | Excluded by title |
| 58 | Sikora M, Goschorska M, Baranowska-Bosiacka I, Chlubek D. In Vitro Effect of 3D Plates Used for Surgical Treatment of Condylar Fractures on Prostaglandin E₂ (PGE₂) and Thromboxane B₂ (TXB₂) Concentration in THP-1 Macrophages. Int J Mol Sci. 2017;18(12). | Excluded by title |
| 59 | Sridharan G, Panneerselvam E, Ponvel K, Tarun S, Krishna Kumar Raja VB. Maxillofacial trauma in a pregnant patient: Contemporary management principles with a case report & review of literature. Chin J Traumatol. 2020;23(2):78-83. | Excluded by title |
| 60 | Taourirt, Hanachi A, Hafiz S. [Orthopedic treatment of limited oral opening. Various results in the child]. Arch Odonto Estomatol. 1986;2 Spec No:40-1. | Excluded by title |
| 61 | Thakur R, Shigli AL, Thakur G, Ahuja R. Giving a second thought to brisement force - a case report. J Clin Pediatr Dent. 2015;39(2):120-3. | Excluded by title |
| 62 | Thompson JR. Sequelae of traumatic and pathologic changes: the orthodontist's viewpoint. J Am Dent Assoc. 1971;82(4):862-70. | Excluded by title |
| 63 | Wang R, Liu Y, Wang JH, Baur DA. Effect of interfragmentary gap on the mechanical behavior of mandibular angle fracture with three fixation designs: A finite element analysis. J Plast Reconstr Aesthet Surg. 2017;70(3):360-9. | Excluded by title |
| 64 | Wassef HR, Colletti PM. Nuclear Medicine Imaging in the Dentomaxillofacial Region. Dent Clin North Am. 2018;62(3):491-509. | Excluded by title |
| 65 | Yamashiro T, Okada T, Takada K. Case report: facial asymmetry and early condylar fracture. Angle Orthod. 1998;68(1):85-90. | Excluded by title |
| 66 | Yao S, Zhou J, Li Z. Contrast analysis of open reduction and internal fixation and non-surgical treatment of condylar fracture: a meta-analysis. J Craniofac Surg. 2014;25(6):2077-80. | Excluded by title |
| 67 | Defabianis P. The importance of early recognition of condylar fractures in children: A study of 2 cases. Journal of Orofacial Pain. 2004;18(3):253-60. | Excluded by title |
| 68 | Fiorelli G, Merlo P, Dalstra M, Melsen B. Mandibular repositioning in adult patients - an alternative to surgery? A two-year follow-up. Australasian Orthodontic Journal. 2019;35(1):61-70. | Excluded by title |
| 69 | Franc C, Braye F, Ngotene R, Breton P, Freidel M. Osteosynthesis using intrafocal nailing of low subcondylar fractures in adults. Surgical technics and initial results. Revue de stomatologie et de chirurgie maxillo-faciale. 1997;98 Suppl 1:35-9. | Excluded by title |
| 70 | Gibbons AJ, Khattak O. Self-drilling intermaxillary fixation screws in the closed treatment of a condylar fracture. Journal of Oral and Maxillofacial Surgery. 2007;65(2):357. | Excluded by title |
| 71 | Harkness EM, Thorburn DN. Hemifacial microsomia label questioned. (I). Angle Orthodontist. 1990;60(1):5. | Excluded by title |
| 72 | Harvold EP. Centric relation. A study of pressure and tension systems in bone modeling and mandibular positioning. Dental Clinics of North America. 1975;19(3):473-84. | Excluded by title |
| 73 | Hwang HS, Jiang T, Sun L, Lee KM, Oh MH, Biao Y, et al. Condylar head remodeling compensating for condylar head displacement by orthognathic surgery. Journal of Cranio-Maxillofacial Surgery. 2019;47(3):406-13. | Excluded by title |
| 74 | Joos U. An adjustable bone fixation system for sagittal split ramus osteotomy: Preliminary report. British Journal of Oral and Maxillofacial Surgery. 1999;37(2):99-103. | Excluded by title |
| 75 | Khanna JN, Ramaswami R. Protocol for the management of ankylosis of the temporomandibular joint. British Journal of Oral and Maxillofacial Surgery. 2019;57(10):1113-8. | Excluded by title |
| 76 | Ko IC, Park KS, Shin JM, Baik JS. Visual loss after intraoral local anesthesia for the removal of circumzygomatic and circum-mandibular wires: A case report. Journal of Oral and Maxillofacial Surgery. 2015;73(10):1918.e1-.e6. | Excluded by title |
| 77 | Madsen H. What's new on the dental scene? Browsing through the dental literature. Journal of Orofacial Orthopedics. 2003;64(1):1-5. | Excluded by title |
| 78 | Magliocca KR, Edwards SP, Helman JI. Traumatic Bone Cyst of the Condylar Region: Report of 2 Cases. Journal of Oral and Maxillofacial Surgery. 2007;65(6):1247-50. | Excluded by title |
| 79 | May L, Blatter J, Bize P, Tsoumakidou G, Denys A, Broome M. Percutaneous cryoablation of benign bony tumours of the mandible. British Journal of Oral and Maxillofacial Surgery. 2020;58(1):75-8. | Excluded by title |
| 80 | Meazzini MC, Brusati R, Diner P, Giann E, Lalatta F, Magri AS, et al. The importance of a differential diagnosis between true hemifacial microsomia and pseudo-hemifacial microsomia in the post-surgical long-term prognosis. Journal of Cranio-Maxillofacial Surgery. 2011;39(1):10-6. | Excluded by title |
| 81 | Meazzini MC, Caprioglio A, Garattini G, Lenatti L, Poggio CE. Hemandibular hypoplasia successfully treated with functional appliances: Is it truly hemifacial microsomia? Cleft Palate-Craniofacial Journal. 2008;45(1):50-6. | Excluded by title |
| 82 | Nadershah M. Orthognathic surgery for correction of facial asymmetry after condylar fracture using computer virtual planning: A case report. World Journal of Dentistry. 2020;11(2):156-60. | Excluded by title |
| 83 | Nieblerová J, Foltán R, Hanzelka T, Pavlíková G, Vlk M, Klíma K, et al. Stability of the miniplate osteosynthesis used for sagittal split osteotomy for closing an anterior open bite: An experimental study in mini-pigs. International Journal of Oral and Maxillofacial Surgery. 2012;41(4):482-8. | Excluded by title |
| 84 | Ord RA, Warburton G, Caccamese JF. Osteochondroma of the condyle: review of 8 cases. International Journal of Oral and Maxillofacial Surgery. 2010;39(6):523-8. | Excluded by title |
| 85 | Pirttiniemi P, Kantomaa T, Sorsa T. Effect of decreased loading on the metabolic activity of the mandibular condylar cartilage in the rat. European Journal of Orthodontics. 2004;26(1):1-5. | Excluded by title |
| 86 | Ramsay-Baggs P. The Ulster hook for intermaxillary fixation. British Journal of Oral and Maxillofacial Surgery. 2011;49(4):324-5. | Excluded by title |
| 87 | Schneider M, Eckelt U, Reitemeier B, Meissner H, Richter G, Loukota R, et al. Stability of fixation of diacapitular fractures of the mandibular condylar process by ultrasound-aided resorbable pins (SonicWeld Rx® System) in pigs. British Journal of Oral and Maxillofacial Surgery. 2011;49(4):297-301. | Excluded by title |
| 88 | Segami N, Nishimura T, Miyaki K, Adachi H. Tethering technique using bone screws and wire for chronic mandibular dislocation: a preliminary study of refractory cases. International Journal of Oral and Maxillofacial Surgery. 2018;47(8):1065-9. | Excluded by title |
| 89 | Daniels JSM, Ali I. Post-traumatic bifid condyle associated with temporomandibular joint ankylosis: Report of a case and review of the literature. Oral Surgery Oral Medicine Oral Pathology Oral Radiology and Endodontology. 2005;99(6):682-8. | Excluded by title |
| 90 | Dantas DB, Andrade MGS, Marchionni AM. Retromandibular approach to condyle fractures: Two case reports. Cranio-the Journal of Craniomandibular & Sleep Practice. 2007;25(4):292-6. | Excluded by title |
| 91 | Espinosa-Femenia M, Sartorres-Nieto M, Berini-Ayes L, Gay-Escoda C. Bilateral bird mandibular condyle: Case report and literature review. Cranio-the Journal of Craniomandibular & Sleep Practice. 2006;24(2):137-40. | Excluded by title |
| 92 | Fontecha BJ, Fernandez M, Reig LR, Sanchez-Ferrin P, Leist A. Bilateral otorrhagia due to a casual fall. Journal of the American Geriatrics Society. 2002;50(7):1314-5. | Excluded by title |
| 93 | Franc C, Braye F, Ngotene R, Breton P, Freidel M. Surgical treatment of low condylar process fractures using a Kirschner pin in adults: Operative procedure and preliminary results. Revue de Stomatologie et de Chirurgie Maxillo-Faciale. 1997;98(SUPPL. 1):35-9. | Excluded by title |
| 94 | Fu K-Y, Li Y-W, Zhang Z-K, Ma X-C. Osteonecrosis of the mandibular condyle as a precursor to osteoarthrosis: A case report. Oral Surgery Oral Medicine Oral Pathology Oral Radiology and Endodontology. 2009;107(1):E34-E8. | Excluded by title |
| 95 | Gaillard A, Nicouleau P, Courtay D, Goichon P. Use of a Kirschner Nail in Treatment of Low Sub-Condylar Fractures of Mandible. Revue De Stomatologie Et De Chirurgie Maxillo-Faciale. 1976;77(8):961-70. | Excluded by title |
| 96 | Gomes MB, Guimaraes SMR, Filho RG, Neves ACC. Traumatic fractures of the tympanic plate: A literature review and case report. Cranio-the Journal of Craniomandibular & Sleep Practice. 2007;25(2):134-7. | Excluded by title |
| 97 | Guelicher D, Gerlach KL. Failed closed reduction of a bifocal mandibular fracture because of dislocation of the mandibular ramus behind the styloid: Case report. British Journal of Oral and Maxillofacial Surgery. 2000;38(4):280-2. | Excluded by title |
| 98 | Gupta B, Acharya A, Singh S, Brazzoli S, Ghorab M, Malik S, et al. Evaluation of jawbone morphology and bone density indices in panoramic radiographs of selective serotonin reuptake inhibitor users: a preliminary study. Dentomaxillofacial Radiology. 2019;48(1). | Excluded by title |
| 99 | Hara S, Mitsugi M, Kanno T, Tatemoto Y. Clinical Approach for Mandibular Advancement by Intraoral Vertical Ramus Osteotomy With Endoscopically Assisted Intraoral Fixation of an L-Shaped Compact Lock Plate. Journal of Craniofacial Surgery. 2013;24(2):545-7. | Excluded by title |
| 100 | Harstall R, Gratz KW, Zwahlen RA. Mandiibular condyle dislocation into the middle cranial fossa: A case report and review of literature. Journal of Trauma-Injury Infection and Critical Care. 2005;59(6):1495-503. | Excluded by title |
| 101 | Heo YJ, Song HC, Kim SM, Bom HS. Time-related change of three-phase Tc-99m MDP bone scintigraphy in patients with thoraco-lumabar compression fractures. Journal of Nuclear Medicine. 2003;44(5):149P-P. | Excluded by title |
| 102 | Hsieh C-H, Chen C-T, Tsai H-H, Lai J-P. Lateral dislocation of bilateral intact mandibular condyles with symphysis fracture: A case report. Journal of Trauma-Injury Infection and Critical Care. 2007;62(6):1518-21. | Excluded by title |
| 103 | Iida S, Nomura K, Okura M, Kogo M. Influence of the incompletely erupted lower third molar on mandibular angle and condylar fractures. Journal of Trauma-Injury Infection and Critical Care. 2004;57(3):613-7. | Excluded by title |
| 104 | Kroetsch LJ, Brook AL, Kader A, Eisig SB. Traumatic dislocation of the mandibular condyle into the middle cranial fossa: Report of a case, review of the literature, and a proposal management protocol. Journal of Oral and Maxillofacial Surgery. 2001;59(1):88-94. | Excluded by title |
| 105 | Kromka-Szydek M, Jedrusik-Pawlowska M, Milewski G, Lekston Z, Cieslik T, Drugacz J. Numerical analysis of displacements of mandible bone parts using various elements for fixation of subcondylar fractures. Acta of Bioengineering and Biomechanics. 2010;12(1):11-8. | Excluded by title |
| 106 | Langton SG, Saeed SR, Musgrove BT, Ramsden RT. Deafness and cholesteatoma complicating fracture of the mandibular condyle. British Journal of Oral & Maxillofacial Surgery. 1996;34(4):286-8. | Excluded by title |
| 107 | Learreta JA, Matos JLF, Freire Matos M, Durst AC. Current Diagnosis of Temporomandibular Pathologies. Cranio-the Journal of Craniomandibular & Sleep Practice. 2009;27(2):125-33. | Excluded by title |
| 108 | Lee C, Stiebel M, Young DM. Cranial nerve VII region of the traumatized facial skeleton: Optimizing fracture repair with the endoscope. Journal of Trauma-Injury Infection and Critical Care. 2000;48(3):423-31. | Excluded by title |
| 109 | Lineaweaver W, Vargervik K, Tomer BS, Ousterhout DK. Posttraumatic Condylar Hyperplasia. Annals of Plastic Surgery. 1989;22(2):163-72. | Excluded by title |
| 110 | Liu P, Li F, Peng L. Treating of old dislocation of mandibular condyle into the middle cranial fossa by gap arthoroplasty combined with temporalis myofascial flap implanting: a case report. International Journal of Clinical and Experimental Medicine. 2017;10(9):13850-5. | Excluded by title |
| 111 | Liu Y, Bai N, Song G, Zhang X, Hu J, Zhu S, et al. Open versus closed treatment of unilateral moderately displaced mandibular condylar fractures: a meta-analysis of randomized controlled trials. Oral Surgery Oral Medicine Oral Pathology Oral Radiology. 2013;116(2):169-73. | Excluded by title |
| 112 | Liu Y-f, Fan Y-y, Jiang X-f, Baur DA. A customized fixation plate with novel structure designed by topological optimization for mandibular angle fracture based on finite element analysis. Biomedical Engineering Online. 2017;16. | Excluded by title |
| 113 | Liu Y-f, Wang R, Baur DA, Jiang X-f. A finite element analysis of the stress distribution to the mandible from impact forces with various orientations of third molars. Journal of Zhejiang University-Science B. 2018;19(1):38-48. | Excluded by title |
| 114 | Magge SN, Chen HI, Heuer GG, Carrasco LR, Storm PB. Dislocation of the mandible into the middle cranial fossa - Case report. Journal of Neurosurgery. 2007;107(1):75-8. | Excluded by title |
| 115 | Matsuda S, Yoshimura H, Kondo S, Sano K. Temporomandibular dislocation caused by pancreatic cancer metastasis: A case report. Oncology Letters. 2017;14(5):6053-8. | Excluded by title |
| 116 | Mellor TK, Shaw RJ. Frey's syndrome following fracture of the mandibular condyle: Case report and literature review. Injury-International Journal of the Care of the Injured. 1996;27(5):359-60. | Excluded by title |
| 117 | Meningaud JP, Maladieere E, Bado F, Bertrand JC, Guilbert F. Eckelt's technique: Evaluation of the access route used at the Salpetriere hospital. Revue de Stomatologie et de Chirurgie Maxillo-Faciale. 1999;100(3):107-10. | Excluded by title |
| 118 | Mishima S, Takahashi K, Hori M, Komatani T, Yamada J, Bessho K. Perioperative Management of Mandibular Fractures in a Patient with Severe Hyperthyroidism: A Case Report. Journal of Musculoskeletal Research. 2016;19(4):1672003-Article No.: . | Excluded by title |
| 119 | Moshy J, Mosha HJ, Lema PA. Prevalence of maxillo-mandibular fractures in Mainland Tanzania. East African Medical Journal. 1996;73(3):172-5. | Excluded by title |
| 120 | Muth RO. [Splinting a condylar fracture by means of an activator and a temporary headgear with chin cap]. Die Quintessenz. 1973;24(6):17-8. | Excluded by title |
| 121 | Nagori SA, Jose A, Bhutia O, Roychoudhury A. Undiagnosed mandibular condylar fractures causing temporomandibular joint ankylosis: A problem in northern India. National Medical Journal of India. 2014;27(5):251-5. | Excluded by title |
| 122 | Oji C. Jaw fractures in Enugu, Nigeria, 1985-95. British Journal of Oral & Maxillofacial Surgery. 1999;37(2):106-9. | Excluded by title |
| 123 | Piagkou M, Tzika M, Paraskevas G, Natsis K. Anatomic variability in the relation between the retromandibular vein and the facial nerve: a case report, literature review and classification. Folia Morphologica. 2013;72(4):371-5. | Excluded by title |
| 124 | Salisbury SK, Cantwell HD. Conservative Management of Fractures of the Mandibular Condyloid Process in 3 Cats and One Dog. Journal of the American Veterinary Medical Association. 1989;194(1):85-7. | Excluded by title |
| 125 | Sandler NA, Andreasen KH, Johns FR. The use of endoscopy in the management of subcondylar fractures of the mandible - A cadaver study. Oral Surgery Oral Medicine Oral Pathology Oral Radiology and Endodontics. 1999;88(5):529-31. | Excluded by title |
| 126 | Santos TdS, Albuquerque KM, Sousa Maciel Santos ME, Laureano Filho JR. Survey on Complications of Orthognathic Surgery Among Oral and Maxillofacial Surgeons. Journal of Craniofacial Surgery. 2012;23(5):E423-E30. | Excluded by title |
| 127 | Scafati CT, Aliberti F, di Clemente SS, Scafati ST, Facciuto E, Cinalli G. Dislocation of a fractured mandibular condyle into the middle cranial fossa: a case treated by an extracranial approach. Childs Nervous System. 2008;24(9):1067-70. | Excluded by title |
| 128 | Schoen R, Gellrich NC, Schmelzeisen R. Minimally invasive open reduction of a displaced condylar fracture in a child. British Journal of Oral & Maxillofacial Surgery. 2005;43(3):258-60. | Excluded by title |
| 129 | Schuller-Gotzburg P, Krenkel C, Reiter TJ, Plenk H. 2D-finite element analyses and histomorphology of lag screws with and without a biconcave washer. Journal of Biomechanics. 1999;32(5):511-20. | Excluded by title |
| 130 | Singh V, Kshirsagar R, Halli R, Sane V, Chhabaria G, Ramanojam S, et al. Evaluation of bioresorbable plates in condylar fracture fixation: a case series. International Journal of Oral and Maxillofacial Surgery. 2013;42(12):1503-5. | Excluded by title |
| 131 | Soos B, Janovics K, Toth A, Szalma J. The role of occlusal support and lower third molars in mandibular angle and condylar fractures. Orvosi Hetilap. 2020;161(28):1166-74. | Excluded by title |
| 132 | Takahashi T, Homma H, Nagai H, Seki H, Kondoh T, Yamazaki Y, et al. Specific expression of inducible nitric oxide synthase in the synovium of the diseased temporomandibular joint. Oral Surgery Oral Medicine Oral Pathology Oral Radiology and Endodontics. 2003;95(2):174-81. | Excluded by title |
| 133 | Vanhove F, Dom M. Zygomatico-coronoid ankylosis: a case report. International Journal of Oral and Maxillofacial Surgery. 1999;28(4):258-9. | Excluded by title |
| 134 | Veyssiere A, Leprovost N, Ambroise B, Prevost R, Chatellier A, Benateau H. Study of the mechanical reliability of an S-shaped adjustable osteosynthesis plate for bilateral sagittal split osteotomies. Study on 15 consecutive cases. Journal of Stomatology Oral and Maxillofacial Surgery. 2018;119(1):19-24. | Excluded by title |
| 135 | Wasserburger M, Edwards PC, Saini TS, Norton NS. A comparison of dental WAT scans and routine histological slides in observing osteoarthritic changes in the mandibular condyles of cadavers. Faseb Journal. 2007;21(5):A599-A600. | Excluded by title |
| 136 | West JL, Palma AE, Vilella L, Fargen KM, Branch CL, Wolfe SQ. Occipital Condyle Fractures and Concomitant Cervical Spine Fractures: Implications for Management. World Neurosurgery. 2018;115:E238-E43. | Excluded by title |
| 137 | Wilmot JJ, Chiego DJ, Carlson DS, Hanks CT, Moskwa JJ. Autoradiographic Study of the Effects of Pulsed Electromagnetic-Fields on Bone and Cartilage Growth in Juvenile Rats. Archives of Oral Biology. 1993;38(1):67-74. | Excluded by title |
| 138 | Xiao E, Li JM, Yan YB, An JG, Duan DH, Gan YH, et al. Decreased Osteogenesis in Stromal Cells from Radiolucent Zone of Human TMJ Ankylosis. Journal of Dental Research. 2013;92(5):450-5. | Excluded by title |
| 139 | Yan J, Liu W, Lin R, Ye M. Incidence and risk factors of the temporomandibular joint disorders in the patients without condylar fractures. M S-Medecine Sciences. 2018;34:39-42. | Excluded by title |
| 140 | Yura S, Ohga N, Ooi K, Izumiyama Y. Intra-Articular Fracture of the Mandibular Condyle: A Case Report. Cranio-the Journal of Craniomandibular & Sleep Practice. 2012;30(3):227-30. | Excluded by title |
| 141 | Zhang M, Alexander AL, Most SP, Li G, Harris OA. Intracranial Dislocation of the Mandibular Condyle: A Case Report and Literature Review. World Neurosurgery. 2016;86. | Excluded by title |
| 142 | Do-Gyoon K, Yong-Hoon J, Cheol-Min H, Amanda AM. Elastic and viscoelastic properties associate with oral bone fracture at the tissue- and macro-levels. Journal of Orthopaedic Research. 2016;34. | Excluded by title |
| 143 | Goel A, Sabat D, Agrawal P. Arthroscopic-assisted fixation of Hoffa fracture: A case report and description of technique. Journal of Clinical Orthopaedics and Trauma. 2016;7(1):61-5. | Excluded by title |
| 144 | Hanson T, Redfern R. Polycystic ovarian syndrome (PCOS), headache and temporomandibular degeneration (TMJ); a case study. Archives of Physical Medicine and Rehabilitation. 2016;97(10):e111. | Excluded by title |
| 145 | Hira PG, Rikhotso RE. Superolateral extracapsular dislocation of the mandibular condyle: Review of the literature and report of two cases. Oral and Maxillofacial Surgery Cases. 2019;5(1). | Excluded by title |
| 146 | Jyothiprasanth M, JeyaVenkatesh P, Khan PS, Gopakumar TS. Arthroscopy-assisted tibial plateau fracture surgery - Minimum 2 years follow up results. Journal of Arthroscopy and Joint Surgery. 2020;7(2):74-7. | Excluded by title |
| 147 | Kaplan RG. Hemifacial microsomia label questioned (I: reply). Angle Orthodontist. 1990;60(1):6. | Excluded by title |
| 148 | D´Andrea AV, Medina AC, Martínez MG, Da Silva L. Tratamento conservador das fraturas de côndilo mandibular em crianças: Relatório de casos. Rev odontopediatr latinoam. 2017;7(2):128-43. | Excluded by title |
| 149 | Kobayashi Y, Satoh K, Mizutani H. Osteogenesis Imperfecta Diagnosed from Mandibular and Lower Limb Fractures: A Case Report. Craniomaxillofacial Trauma and Reconstruction. 2016;9(2):141-4. | Excluded by title |
| 150 | Kulahci Y, Sever C, Uygur F, Guney Senol M, Cayci T. Mandible fractures during epileptic seizure: Two case reports. European Journal of Plastic Surgery. 2009;32(5):253-5. | Excluded by title |
| 151 | Kurita K. Recurrent mandibular dislocation: Future prospects and trials for elderly patients. International Journal of Oral and Maxillofacial Surgery. 2017;46:31. | Excluded by title |
| 152 | Landreau P, Catteeuw A, Hamie F, Saithna A, Sonnery-Cottet B, Smigielski R. Anatomic Study and Reanalysis of the Nomenclature of the Anterolateral Complex of the Knee Focusing on the Distal Iliotibial Band: Identification and Description of the Condylar Strap. Orthopaedic Journal of Sports Medicine. 2019;7(1). | Excluded by title |
| 153 | Leal MOCD, Teixeira RG, Jodas CRP, Gabarra FR, Moraes PC, Júnior WZ, et al. Multiple face fractures with severe joint complication: case report. International Journal of Oral and Maxillofacial Surgery. 2019;48:141. | Excluded by title |
| 154 | Makino K, Hirano A, Shidayama R, Fujii T. Corrective surgery for patients with temporomandibular ankylosis with severe micrognathia: Results in three cases. Japanese Journal of Plastic and Reconstructive Surgery. 1996;39(11):1129-38. | Excluded by title |
| 155 | Mohanty S, Gulati U, Kathuria S. Pseudoaneurysm of the internal maxillary artery: A rare complication of condylar fracture. Craniomaxillofacial Trauma and Reconstruction. 2013;6(4):271-4. | Excluded by title |
| 156 | Nardi C, Vignoli C, Pietragalla M, Tonelli P, Calistri L, Franchi L, et al. Imaging of mandibular fractures: a pictorial review. Insights into Imaging. 2020;11(1). | Excluded by title |
| 157 | Park MW, Eo MY, Seo BY, Nguyen TTH, Kim SM. Gap arthroplasty with active mouth opening exercises using an interocclusal splint in temporomandibular joint ankylosis patients. Maxillofacial and Plastic Reconstructive Surgery. 2019;41(1). | Excluded by title |
| 158 | Pereira FL, Pinheiro LDMDA, Araújo PM, Chihara LL, Maia Nogueira RL, Sant'Ana E. Surgical Treatment of Posttraumatic Laterognathia: A Case Report and a Literature Review, Focused on the Effects of a Condylar Fracture on the Face. Craniomaxillofacial Trauma and Reconstruction. 2018;11(3):211-8. | Excluded by title |
| 159 | Ribeiro-Junior PD, Padovan LEM, Momesso NR, Duarte GLC, Ficho AC. Non-surgical management of bilateral mandibular condyle fracture: Six-year follow-up. A case report. Revista Portuguesa de Estomatologia, Medicina Dentaria e Cirurgia Maxilofacial. 2018;59(1):44-8. | Excluded by title |
| 160 | Singh PK. Osteoarticular sequlae of small pox: A case report. Asian Pacific Journal of Tropical Medicine. 2010;3(12):1001-3. | Excluded by title |
| 161 | Singh PK. Osteomyelitis variolosa with fracture: A unique case report. Annals of Tropical Medicine and Public Health. 2012;5(2):124-6. | Excluded by title |
| 162 | Bencini C, Scollo SB. Fractura de cuello de cóndilo en paciente con crecimiento. Ortodoncia. 2002;66(132):60-7. | Excluded by title |
| 163 | Dantas RMX, Malaquias PTIA, Spínola LG, Costa MVOC, Oliveira GQV, Azevedo RAd. Tratamento conservador de fratura condilar por projétil de arma de fogo: relato de caso. Rev Odontol Araçatuba (Online). 2013;34(1):71-4. | Excluded by title |
| 164 | Navarro RdL. Avaliação molecular, tomográfica e microscópica dos côndilos após avanço cirúrgico mandibular utilizando fixação rígida e semi-rígida: estudo em minipigs. 2007. p. xxi,117-xxi,. | Excluded by title |
| 165 | Rivas N, Lorenz G, Ceccarelli A. Las posibilidades de la ortopedia funcional de los maxilares: rehabilitación de un paciente con secuela de fractura de cóndilo: laterodesviación esqueletal. Investig docencia. 2002;3(6):28-9. | Excluded by title |
| 166 | Rossi EG. Características y confección de un distractor RA. DI. CA. Rev Asoc Argent Ortop Funcional Maxilares. 2014;40(1):49-52. | Excluded by title |
| 167 | Salgado CV, Jung A, Ferraz CL, Jorge WA. Tratamento ortopédico-funcional das fraturas do côndilo mandibular em crianças: relato de casos. RPG rev pos-grad. 1995;2(4):224-30. | Excluded by title |
| 168 | Stevão ÉLdL. Teste biomecânico de arrancamento de mini âncora inserida em osso bovino û descrição de dispositivos e técnicas. Full dent sci. 2016;7(25):149-55. | Excluded by title |
| 169 | Travagin MWC, Tocolini DG, Wilhelmsen NSW, Deantoni C. Tratamento de fratura bilateral da cabeça da mandíbula com o uso de aparelho protrator mandibular: relato de caso. Rev clín ortodon Dental Press. 2013;12(4):57-64. | Excluded by title |
| 170 | Turcio KHL, Silva EVF, Laurindo Junior MCB, Bonatto LdR, Nagay BE, Guiotti AM, et al. Fratura de côndilo mandibular não tratada e disfunção temporomandibular: relato de caso. Rev Odontol Araçatuba (Impr). 2017;38(1):46-51. | Excluded by title |
| 171 | Valladares-Pérez S, Bustamante-Correa D, Sepúlveda-Troncoso G. Tratamiento Ortopédico de las Fracturas de Cóndilo Mandibular A Propósito de Tres Casos. Int j odontostomatol (Print). 2019;13(2):157-61. | Excluded by title |
| 172 | Almeida LE, Baioni CS, Martins AP, Line SR, Noronha L, Trevilatto PC, et al. Histologic and histomorphometric analysis of posterior region of the human temporomandibular disc. Oral Surg Oral Med Oral Pathol Oral Radiol Endod. 2008;105(4):e6-11. | Excluded by abstract |
| 173 | Andreas ZJ, Benoit S, Olivier L, Nikola S, Hanna T, Tateyuki I. Incidence, aetiology and pattern of mandibular fractures in central Switzerland. Swiss Medical Weekly. 2011;141. | Excluded by abstract |
| 174 | Armond ACV, Martins CC, Glória JCR, Galvão EL, Dos Santos CRR, Falci SGM. Influence of third molars in mandibular fractures. Part 2: mandibular condyle-a meta-analysis. Int J Oral Maxillofac Surg. 2017;46(6):730-9. | Excluded by abstract |
| 175 | Barron RP, Kainulainen VT, Gusenbauer AW, Hollenberg R, Sandor GKB. Fracture of glenoid fossa and traumatic dislocation of mandibular condyle into middle cranial fossa. Oral Surgery Oral Medicine Oral Pathology Oral Radiology and Endodontics. 2002;93(6):640-2. | Excluded by abstract |
| 176 | Boole JR, Holtel M, Amoroso P, Yore M. 5196 mandible fractures among 4381 active duty army soldiers, 1980 to 1998. Laryngoscope. 2001;111(10):1691-6. | Excluded by abstract |
| 177 | Carinci F, Arduin L, Pagliaro F, Zollino I, Brunelli G, Cenzi R. Scoring Mandibular Fractures: A Tool for Staging Diagnosis, Planning Treatment, and Predicting Prognosis. Journal of Trauma-Injury Infection and Critical Care. 2009;66(1):215-9. | Excluded by abstract |
| 178 | Cousley RR, Gibbons AJ. Correction of the occlusal and functional sequelae of mandibular condyle fractures using orthodontic mini-implant molar intrusion. J Orthod. 2014;41(3):245-53. | Excluded by abstract |
| 179 | Acebal-Bianco F, Vuylsteke PL, Mommaerts MY, De Clercq CA. Perioperative complications in corrective facial orthopedic surgery: a 5-year retrospective study. J Oral Maxillofac Surg. 2000;58(7):754-60. | Excluded by abstract |
| 180 | Basdra EK, Stellzig A, Komposch G. Functional treatment of condylar fractures in adult patients. Am J Orthod Dentofacial Orthop. 1998;113(6):641-6. | Excluded by abstract |
| 181 | Chatzistavrou EK, Basdra EK. Conservative treatment of isolated condylar fractures in growing patients. World J Orthod. 2007;8(3):241-8. | Excluded by abstract |
| 182 | Cole P, Kaufman Y, Izaddoost S, Hatef DA, Hollier L. Principles of pediatric mandibular fracture management. Plast Reconstr Surg. 2009;123(3):1022-4. | Excluded by abstract |
| 183 | Colella G, Corvo G, Napolitano A, Tartato GP, Longobardi G. [The functional therapy of fractures of the mandibular condyle. Our experience]. Minerva Stomatol. 1996;45(9):393-9. | Excluded by abstract |
| 184 | Conte R, Forin Valvecchi F, Gracco AL, Bruno G, De Stefani A. Condylar dysfunctional remodeling and recortication: a case-control study. Minerva Stomatol. 2019;68(2):74-83. | Excluded by abstract |
| 185 | Croce A, Moretti A, Vitullo F, Castriotta A, Rosa de M, Citraro L. Transparotid approach for mandibular condylar neck and subcondylar fractures. Acta Otorhinolaryngol Ital. 2010;30(6):303-9. | Excluded by abstract |
| 186 | Darwich MA, Albogha MH, Abdelmajeed A, Darwich K. Assessment of the Biomechanical Performance of 5 Plating Techniques in Fixation of Mandibular Subcondylar Fracture Using Finite Element Analysis. J Oral Maxillofac Surg. 2016;74(4):794.e1-8. | Excluded by abstract |
| 187 | de Oliveira PA, Jr., Pires LF, Oliveira GS, Faber PA. Temporomandibular joint ankylosis after condylar fracture with penetration of the condyle in the medium cranial fossa. J Oral Maxillofac Surg. 2005;63(12):1778-81. | Excluded by abstract |
| 188 | Defabianis P. Condylar fractures treatment in children and youths: influence on function and face development (a five year retrospective analysis). Funct Orthod. 2001;18(2):24-31. | Excluded by abstract |
| 189 | DeFabianis P. Rational and philosophic basis for a functional approach to TMJ fractures in children. Funct Orthod. 2000;17(3):20-4. | Excluded by abstract |
| 190 | Defabianis P. TMJ fractures in children: importance of functional activation of muscles in preventing mandibular asymmetries and facial maldevelopment. Funct Orthod. 2002;19(2):34-42. | Excluded by abstract |
| 191 | Deffez JP, Bordais P, Gros F. [Jaw block at maximal mouth aperture in subcondylar fractures in the very young child]. Rev Stomatol Chir Maxillofac. 1975;76(6):429-42. | Excluded by abstract |
| 192 | Deffez JP, Themar P, Allain P, Berrada K, Bordais P, Brethaux J, et al. [Dynamic bite block in fractures of the mandibular condyle and following surgery of temporomandibular ankylosis in children]. Rev Stomatol Chir Maxillofac. 1991;92(2):65-70. | Excluded by abstract |
| 193 | Derfoufi L, Delaval C, Goudot P, Yachouh J. Complications of condylar fracture osteosynthesis. J Craniofac Surg. 2011;22(4):1448-51. | Excluded by abstract |
| 194 | Ellis E, 3rd, McFadden D, Simon P, Throckmorton G. Surgical complications with open treatment of mandibular condylar process fractures. J Oral Maxillofac Surg. 2000;58(9):950-8. | Excluded by abstract |
| 195 | Ellis E, 3rd, Simon P, Throckmorton GS. Occlusal results after open or closed treatment of fractures of the mandibular condylar process. J Oral Maxillofac Surg. 2000;58(3):260-8. | Excluded by abstract |
| 196 | Farronato G, Grillo ME, Giannini L, Farronato D, Maspero C. Long-term results of early condylar fracture correction: case report. Dent Traumatol. 2009;25(3):e37-42. | Excluded by abstract |
| 197 | Fiorelli G, Merlo P, Dalstra M, Melsen B. [Mandibular repositioning in adult patients. An alternative to surgery in some patients? A two-year follow-up]. Orthod Fr. 2018;89(2):123-35. | Excluded by abstract |
| 198 | Frapier L, Garcia C, Pic E, Morant F, Belguendouz S, Gauthier A, et al. Successful orthodontic-surgical treatment: aiming for esthetics and function. Analysis of some clinical cases. Int Orthod. 2013;11(4):357-88. | Excluded by abstract |
| 199 | Gašpar G, Brakus I, Kovačić I. Conservative orthodontic treatment of mandibular bilateral condyle fracture. J Craniofac Surg. 2014;25(5):e488-90. | Excluded by abstract |
| 200 | Gasparini G, Boniello R, Moro A, Di Nardo F, Pelo S. Orthognathic surgery: a new preoperative informed consent model. J Craniofac Surg. 2009;20(1):90-2. | Excluded by abstract |
| 201 | Hackett JF, Sleeman DJ. Vertical-split fracture of mandibular condyle and its sequelae. Br Dent J. 2001;191(10):557-8. | Excluded by abstract |
| 202 | Hakim SG, Trankle T, Kimmerle H, Sieg P, Jacobsen HC. A new non-endoscopic intraoral approach for open reduction and internal fixation of subcondylar fractures of the mandible. J Craniomaxillofac Surg. 2014;42(7):1166-70. | Excluded by abstract |
| 203 | He D, Yang C, Chen M, Bin J, Zhang X, Qiu Y. Modified preauricular approach and rigid internal fixation for intracapsular condyle fracture of the mandible. J Oral Maxillofac Surg. 2010;68(7):1578-84. | Excluded by abstract |
| 204 | He D, Yang C, Chen M, Jiang B, Wang B. Intracapsular condylar fracture of the mandible: our classification and open treatment experience. J Oral Maxillofac Surg. 2009;67(8):1672-9. | Excluded by abstract |
| 205 | Hirjak D, Galis B, Beno M, Machon V, Mercuri LG, Neff A. Intraoperative arthroscopy of the TMJ during surgical management of condylar head fractures: A preliminary report. J Craniomaxillofac Surg. 2018;46(12):1989-95. | Excluded by abstract |
| 206 | Hjorth T, Melsen B, Møller E. Masticatory muscle function after unilateral condylar fractures: a prospective and quantitative electromyographic study. Eur J Oral Sci. 1997;105(4):298-304. | Excluded by abstract |
| 207 | Hlawitschka M, Eckelt U. Assessment of patients treated for intracapsular fractures of the mandibular condyle by closed techniques. J Oral Maxillofac Surg. 2002;60(7):784-91; discussion 92. | Excluded by abstract |
| 208 | Holtgrave E, Rösli A, Spiessl B. [The treatment of collum fractures in children, clinical and radiographic results]. Dtsch Zahnarztl Z. 1975;30(3):213-21. | Excluded by abstract |
| 209 | Hovinga J, Kraal ER, Roorda LA. A follow-up of osteotomies for dysgnathia. J Maxillofac Surg. 1979;7(4):271-82. | Excluded by abstract |
| 210 | Ishihara Y, Kuroda S, Nishiyama A, Sasaki A, Takano-Yamamoto T, Yamashiro T. Functional improvements after orthodontic-surgical reconstruction in a patient with multiple maxillofacial fractures. Am J Orthod Dentofacial Orthop. 2012;142(4):534-45. | Excluded by abstract |
| 211 | Iwai T, Matsui Y, Omura S, Tohnai I. Endoscopic hemostasis with an ultrasonically activated device for hemorrhage from a branch of the maxillary artery during endoscopically assisted reduction of condylar neck fracture. J Craniofac Surg. 2013;24(2):534-5. | Excluded by abstract |
| 212 | Jabłoński M, Masierek A, Pogoda M. [Use of an apparatus of the authors' design in the orthodontic treatment of fractures of the mandibular condyles]. Czas Stomatol. 1980;33(11):1005-9. | Excluded by abstract |
| 213 | Jing J, Han Y, Song Y, Wan Y. Surgical treatment on displaced and dislocated sagittal fractures of the mandibular condyle. Oral Surg Oral Med Oral Pathol Oral Radiol Endod. 2011;111(6):693-9. | Excluded by abstract |
| 214 | Jones JK, Van Sickels JE. A preliminary report of arthroscopic findings following acute condylar trauma. J Oral Maxillofac Surg. 1991;49(1):55-60. | Excluded by abstract |
| 215 | Joos U. [The development of the treatment of collum fractures in childhood]. Dtsch Zahnarztl Z. 1991;46(1):38-40. | Excluded by abstract |
| 216 | Kawase-Koga Y, Mori Y, Hoshi K, Takato T. A novel technique for preventing skin pressure sores using a rubber tube during surgical treatment of mandibular condyle fractures. J Craniofac Surg. 2013;24(6):e604-6. | Excluded by abstract |
| 217 | Kim MJ, Seo J, Kim DK, Baek SH. Three-dimensional virtual-surgery simulation-assisted asymmetric bilateral mandibular distraction osteogenesis for a patient with bilateral condylar fractures. Am J Orthod Dentofacial Orthop. 2017;151(1):186-200. | Excluded by abstract |
| 218 | Kim YH, Youn S, Kim JT. Early complete bone union after condylar fracture in a child. J Craniofac Surg. 2011;22(4):1516-7. | Excluded by abstract |
| 219 | Klein C, Howaldt HP. Lengthening of the hypoplastic mandible by gradual distraction in childhood--a preliminary report. J Craniomaxillofac Surg. 1995;23(2):68-74. | Excluded by abstract |
| 220 | Kleinheinz J, Anastassov GE, Joos U. Indications for treatment of subcondylar mandibular fractures. J Craniomaxillofac Trauma. 1999;5(2):17-23; discussion 4-6. | Excluded by abstract |
| 221 | Ko EW, Huang CS, Chen YR, Figueroa AA. Cephalometric craniofacial characteristics in patients with temporomandibular joint ankylosis. Chang Gung Med J. 2005;28(7):456-66. | Excluded by abstract |
| 222 | Konaş E, Tunçbilek G, Kayikçioğlu A, Akcan CA, Kocadereli I, Mavili ME. Splint-assisted reduction of comminuted or complex maxillofacial fractures. J Craniofac Surg. 2011;22(4):1471-5. | Excluded by abstract |
| 223 | Koolstra JH, Kommers SC, Forouzanfar T. Biomechanical analysis of fractures in the mandibular neck (collum mandibulae). J Craniomaxillofac Surg. 2014;42(8):1789-94. | Excluded by abstract |
| 224 | Koubayashi S, Nakagawa M, Shimomura T, Susami R. [Repositioner of external fragment of mandibular ramus (ramus repositioner) in sagittal split ramus osteotomy of mandible]. Nihon Kyosei Shika Gakkai Zasshi. 1989;48(5):553-9. | Excluded by abstract |
| 225 | Koumoura F, Papakosta V. A serious complication in the temporomandibular region due to insufficient follow-up. Orthodontics (Chic). 2011;12(2):134-9. | Excluded by abstract |
| 226 | Krenkel C, Strobl V. [Long-term outcome of functional treatment for high double-sided condylar fractures]. Z Stomatol. 1989;86(7):401-12. | Excluded by abstract |
| 227 | Krenkel C. Axial 'anchor' screw (lag screw with biconcave washer) or 'slanted-screw' plate for osteosynthesis of fractures of the mandibular condylar process. J Craniomaxillofac Surg. 1992;20(8):348-53. | Excluded by abstract |
| 228 | Krennmair S, Winterhalder P, Hunger S, Rupperti S, Holberg C. The Effects of Frontal Trauma on 4 Interforaminal Dental Implants: A 3-Dimensional Finite Element Analysis Comparing Splinted and Unsplinted Implant Configurations. J Oral Maxillofac Surg. 2020;78(6):961-72. | Excluded by abstract |
| 229 | Kuntamukkula S, Sinha R, Tiwari PK, Paul D. Dynamic Stability Assessment of the Temporomandibular Joint as a Sequela of Open Reduction and Internal Fixation of Unilateral Condylar Fracture. J Oral Maxillofac Surg. 2018;76(12):2598-609. | Excluded by abstract |
| 230 | Kuroda S, Tanimoto K, Izawa T, Fujihara S, Koolstra JH, Tanaka E. Biomechanical and biochemical characteristics of the mandibular condylar cartilage. Osteoarthritis Cartilage. 2009;17(11):1408-15. | Excluded by abstract |
| 231 | Lachard J, Guilbert F, Gola R, Blanc JL, Bertrand JC, Rocca A, et al. [Intracranial penetration of the mandibular condyle: report on three cases (author's transl)]. Ann Otolaryngol Chir Cervicofac. 1981;98(10-11):543-6. | Excluded by abstract |
| 232 | Lagvankar SP. A manoeuvre to achieve intermaxillary fixation in fractures of the mandibular condyle with an open bite deformity. Br J Oral Maxillofac Surg. 1990;28(1):62. | Excluded by abstract |
| 233 | Lee JW, Lee YC, Kuo YL. Reappraisal of the surgical strategy in treatment of mandibular condylar fractures. Plast Reconstr Surg. 2010;125(2):609-19. | Excluded by abstract |
| 234 | Lee YS, Yi YJ, Kim YK, Lee NK, Larson BE. Conservative interdisciplinary treatment of a case with multiple facial and condyle fractures. Dent Traumatol. 2017;33(3):226-9. | Excluded by abstract |
| 235 | Lemière E, Sicre A, Vereecke F, Brygo A, Nicola J, Ferri J. [Our physiotherapy treatment of articular fractures of the mandibular condyle]. Rev Stomatol Chir Maxillofac. 2003;104(2):104-6. | Excluded by abstract |
| 236 | Levenets AA, Grigor'ian AS. [The pathogenesis of posttraumatic deformities of the mandible in the growing organism]. Stomatologiia (Mosk). 2000;79(1):20-6. | Excluded by abstract |
| 237 | Lewin A, Evans WG, Booth JL, Howes DG. Constrained and unconstrained postures of the mandible--a break with tradition? Ann Acad Med Singap. 1995;24(1):3-10. | Excluded by abstract |
| 238 | Linder-Aronson S. Early interceptive treatment of asymmetry. Proc Finn Dent Soc. 1991;87(1):159-66. | Excluded by abstract |
| 239 | Lloyd T, Nightingale C, Edler R. The use of vacuum-formed splints for temporary intermaxillary fixation in the management of unilateral condylar fractures. Br J Oral Maxillofac Surg. 2001;39(4):301-3. | Excluded by abstract |
| 240 | Luck O, Harzer W. Early treatment of angle Class II, division 2 in combination with functional therapy of TMJ fracture. J Orofac Orthop. 2001;62(2):157-62. | Excluded by abstract |
| 241 | McLeod NMH, Van Gijn D. Use of ultrasound-activated resorbable sheets and pins in the management of fractures of the condylar neck of the mandible: a case series. Br J Oral Maxillofac Surg. 2018;56(3):182-5. | Excluded by abstract |
| 242 | Medina AC. Functional appliance treatment for bilateral condylar fracture in a pediatric patient. Pediatr Dent. 2009;31(5):432-7. | Excluded by abstract |
| 243 | Melsen B, Bjerregaard J, Bundgaard M. The effect of treatment with functional appliance on a pathologic growth pattern of the condyle. Am J Orthod Dentofacial Orthop. 1986;90(6):503-12. | Excluded by abstract |
| 244 | Minagi S, Sakiya M, Sato T, Matsunaga T, Natsuaki N. Vibrating-traction method for mechanical joint distraction. J Oral Rehabil. 2000;27(8):703-7. | Excluded by abstract |
| 245 | Mitchell L. Displacement of a mandibular canine following fracture of the mandible. Br Dent J. 1993;174(11):417-8. | Excluded by abstract |
| 246 | Motta A, Louro RS, Medeiros PJ, Capelli J, Jr. Orthodontic and surgical treatment of a patient with an ankylosed temporomandibular joint. Am J Orthod Dentofacial Orthop. 2007;131(6):785-96. | Excluded by abstract |
| 247 | Neff A, Chossegros C, Blanc JL, Champsaur P, Cheynet F, Devauchelle B, et al. Position paper from the IBRA Symposium on Surgery of the Head--the 2nd International Symposium for Condylar Fracture Osteosynthesis, Marseille, France 2012. J Craniomaxillofac Surg. 2014;42(7):1234-49. | Excluded by abstract |
| 248 | Oliveira CA, de Lima Pedro R, Antunes LA, de Castro Costa M, Primo LG. Image-based evaluation of facial fractures in a child using computed tomography. Gen Dent. 2012;60(5):e280-2. | Excluded by abstract |
| 249 | Panneerselvam E, Prasad PJ, Balasubramaniam S, Somasundaram S, Raja KV, Srinivasan D. The Influence of the Mandibular Gonial Angle on the Incidence of Mandibular Angle Fracture-A Radiomorphometric Study. J Oral Maxillofac Surg. 2017;75(1):153-9. | Excluded by abstract |
| 250 | Paoli JR, Fabié L, Dodart L, Lauwers F, Boutault F, Fabié M. [Mandibular fractures in sports. Retrospective study of 48 cases]. Rev Stomatol Chir Maxillofac. 1999;100(6):306-10. | Excluded by abstract |
| 251 | Papadaki ME, Tayebaty F, Kaban LB, Troulis MJ. Condylar resorption. Oral Maxillofac Surg Clin North Am. 2007;19(2):223-34, vii. | Excluded by abstract |
| 252 | Papadopoulos K, Tanić T, Mitić V. [Orthodontic management of facial asymmetry caused by early condilar fracture in a growing patient]. Srp Arh Celok Lek. 2012;140(9-10):630-6. | Excluded by abstract |
| 253 | Pedemonte C, Carmona A, González E, Vargas I, Lopetegui F, Rojas E. Correlation Between the Posterior Mandibular Width and the Lingual Gap Caused by Symphyseal Fractures Using a Virtual Model. J Oral Maxillofac Surg. 2018;76(4):832.e1-.e8. | Excluded by abstract |
| 254 | Pepato AO, Palinkas M, Regalo SC, de Medeiros EH, de Vasconcelos PB, Sverzut CE, et al. Effect of surgical treatment of mandibular fracture: electromyographic analysis, bite force, and mandibular mobility. J Craniofac Surg. 2014;25(5):1714-20. | Excluded by abstract |
| 255 | Pirttiniemi P, Peltomäki T, Müller L, Luder HU. Abnormal mandibular growth and the condylar cartilage. Eur J Orthod. 2009;31(1):1-11. | Excluded by abstract |
| 256 | Pogosian Iu M, Badalian Kh A, Sysoeva EN. [The treatment of fractures of the mandibular condylar processes]. Stomatologiia (Mosk). 1991(4):44-7. | Excluded by abstract |
| 257 | Pons J, Pasturel A, Desgeorges M, De Greslan M. [Traumatic intracranial penetration of the mandibular condyle]. Rev Stomatol Chir Maxillofac. 1976;77(6):869-74. | Excluded by abstract |
| 258 | Reuther J. [Orthognathic surgery: corrective bone operations]. Mund Kiefer Gesichtschir. 2000;4 Suppl 1:S237-48. | Excluded by abstract |
| 259 | Roberts WE, Stocum DL. Part II: Temporomandibular Joint (TMJ)-Regeneration, Degeneration, and Adaptation. Curr Osteoporos Rep. 2018;16(4):369-79. | Excluded by abstract |
| 260 | Rönning O. [Compensatory skeletal growth modifications]. Stomatol DDR. 1977;27(1):55-9. | Excluded by abstract |
| 261 | Salo L, Raustia A, Pernu H, Virtanen K. Internal derangement of the temporomandibular joint: a histochemical study. J Oral Maxillofac Surg. 1991;49(2):171-6. | Excluded by abstract |
| 262 | Samieirad S, Khajehahmadi S, Tohidi E, Barzegar M. Unusual Presentation of Guillain-Barré Syndrome After Mandibular Fracture Treatment: A Review of the Literature and a New Case. J Oral Maxillofac Surg. 2016;74(1):129.e1-6. | Excluded by abstract |
| 263 | Sander FG. [Biomechanical aspects of the spring-active-appliance during the night sleep]. Prakt Kieferorthop. 1991;5(1):17-28. | Excluded by abstract |
| 264 | Schneider M, Stadlinger B, Loukota R, Eckelt U. Three-dimensional fixation of fractures of the mandibular condyle with a resorbable three-dimensional osteosynthesis mesh. Br J Oral Maxillofac Surg. 2012;50(5):470-3. | Excluded by abstract |
| 265 | Sforza C, Tartaglia GM, Lovecchio N, Ugolini A, Monteverdi R, Giannì AB, et al. Mandibular movements at maximum mouth opening and EMG activity of masticatory and neck muscles in patients rehabilitated after a mandibular condyle fracture. J Craniomaxillofac Surg. 2009;37(6):327-33. | Excluded by abstract |
| 266 | Shetty V, Niederdellmann H. Maxillomandibular fixation with minihooks: a clinical evaluation. Oral Surg Oral Med Oral Pathol. 1987;64(6):677-9. | Excluded by abstract |
| 267 | Shirrazi M, Ehterami S, Djahanguiri B. Appliance effects on the masseter and temporal muscle electromyography of patients with facial asymmetry. J Pedod. 1990;14(4):240-1. | Excluded by abstract |
| 268 | Silvennoinen U, Iizuka T, Oikarinen K, Lindqvist C. Analysis of possible factors leading to problems after nonsurgical treatment of condylar fractures. J Oral Maxillofac Surg. 1994;52(8):793-9. | Excluded by abstract |
| 269 | Silvestri A, Lattanzi A, Mantuano MT. A protocol for the treatment of mandibular condylar fractures. Minerva Stomatol. 2004;53(7-8):403-15. | Excluded by abstract |
| 270 | Sugiura T, Yamamoto K, Murakami K, Sugimura M. A comparative evaluation of osteosynthesis with lag screws, miniplates, or Kirschner wires for mandibular condylar process fractures. J Oral Maxillofac Surg. 2001;59(10):1161-8; discussion 9-70. | Excluded by abstract |
| 271 | Takano H, Takahashi T, Nakata A, Nogami S, Yusa K, Kuwajima S, et al. Facilitation of bone resorption activities in synovial lavage fluid patients with mandibular condyle fractures. J Oral Rehabil. 2016;43(5):333-9. | Excluded by abstract |
| 272 | Tatsumi H, Nakatani E, Kanno T, Nariai Y, Kagimura T, Sekine J. Clinical Features and Treatment Modes of Mandibular Fracture at the Department of Oral and Maxillofacial Surgery, Shimane University Hospital, Japan. PLoS One. 2015;10(9):e0136278. | Excluded by abstract |
| 273 | Tavares CA, Allgayer S. Conservative orthodontic treatment for a patient with a unilateral condylar fracture. Am J Orthod Dentofacial Orthop. 2012;141(5):e75-84. | Excluded by abstract |
| 274 | Valstar MH, Jaspers GW, de Lange J. [Insufficient diagnostics in a patient with a fracture of the mandibular collum]. Ned Tijdschr Tandheelkd. 2013;120(3):151-3. | Excluded by abstract |
| 275 | Van Bellinghen X, Idoux-Gillet Y, Pugliano M, Strub M, Bornert F, Clauss F, et al. Temporomandibular Joint Regenerative Medicine. Int J Mol Sci. 2018;19(2). | Excluded by abstract |
| 276 | van der Linden WJ. Dislocation of the mandibular condyle into the middle cranial fossa: report of a case with 5 year CT follow-up. Int J Oral Maxillofac Surg. 2003;32(2):215-8. | Excluded by abstract |
| 277 | Wichelhaus A, Haas R, Sander FG, Kreidler JF. The influence of the spring activator on the mobility of the lower jaw in traumatically injured patients. J Orofac Orthop. 1998;59(6):340-51. | Excluded by abstract |
| 278 | Williamson EH. Condylar remodeling and growth following fracture in a child. Facial Orthop Temporomandibular Arthrol. 1987;4(1):3-7. | Excluded by abstract |
| 279 | Williamson EH. Treatment of condylar fracture using a functional appliance in a 6-year-old child. Facial Orthop Temporomandibular Arthrol. 1986;3(8):3-5. | Excluded by abstract |
| 280 | Xu Y, Gong SG, Zhu F, Li M, Biao X. Conservative orthodontic fixed appliance management of pediatric mandibular bilateral condylar fracture. Am J Orthod Dentofacial Orthop. 2016;150(1):181-7. | Excluded by abstract |
| 281 | Yan G, Zhou Q, Yang M. A New Method To Reposition the Displaced Articular Disc For a Patient With Comminuted Condylar Fracture. J Craniofac Surg. 2019;30(4):e373-e6. | Excluded by abstract |
| 282 | Yanagita T, Adachi R, Kamioka H, Yamashiro T. Severe open bite due to traumatic condylar fractures treated nonsurgically with implanted miniscrew anchorage. Am J Orthod Dentofacial Orthop. 2013;143(4 Suppl):S137-47. | Excluded by abstract |
| 283 | Zachariades N, Papavassiliou D, Koumoura F. Fractures of the facial skeleton in children. J Craniomaxillofac Surg. 1990;18(4):151-3. | Excluded by abstract |
| 284 | Zhang QB, Zhang B, Zhang ZQ, Chen Q. The epidemiology of cranio-facial injuries caused by animals in southern-central China. J Craniomaxillofac Surg. 2012;40(6):506-9. | Excluded by abstract |
| 285 | Beziat JL, Ribeiro C, Champsaur A, Freidel M, Dumas P. A critical study of the treatment of mandibular fractures. A one year survey of patients seen at the Clinic of Maxillo-Facial Surgery in Lyon. Revue de Stomatologie et de Chirurgie Maxillo-Faciale. 1982;83(5):273-8. | Excluded by abstract |
| 286 | Chen S, Zhang Y, An JG, He Y. Width-Controlling Fixation of Symphyseal/Parasymphyseal Fractures Associated with Bilateral Condylar Fractures with 2 2.0-mm Miniplates: A Retrospective Investigation of 45 Cases. Journal of Oral and Maxillofacial Surgery. 2016;74(2):315-27. | Excluded by abstract |
| 287 | Cutbirth M, Van Sickels JE, Thrash WJ. Condylar resorption after bicortical screw fixation of mandibular advancement. Journal of Oral and Maxillofacial Surgery. 1998;56(2):178-82. | Excluded by abstract |
| 288 | Dodson TB. Condyle and ramus-condyle unit fractures in growing patients: Management and outcomes. Oral and Maxillofacial Surgery Clinics of North America. 2005;17(4 SPEC. ISS.):447-53. | Excluded by abstract |
| 289 | Eggensperger N, Smolka K, Luder J, Iizuka T. Short- and long-term skeletal relapse after mandibular advancement surgery. International Journal of Oral and Maxillofacial Surgery. 2006;35(1):36-42. | Excluded by abstract |
| 290 | Fluur E. Unusual complication following surgically treated fracture of the mandibular condyle. International Journal of Oral Surgery. 1973;2(6):297-302. | Excluded by abstract |
| 291 | He Y, Zhang Y, Li ZL, An JG, Yi ZQ, Bao SD. Treatment of traumatic dislocation of the mandibular condyle into the cranial fossa: Development of a probable treatment algorithm. International Journal of Oral and Maxillofacial Surgery. 2015;44(7):864-70. | Excluded by abstract |
| 292 | Huang IY, Chen CM, Kao YH, Chen CM, Wu CW. Management of long-standing mandibular dislocation. International Journal of Oral and Maxillofacial Surgery. 2011;40(8):810-4. | Excluded by abstract |
| 293 | Kommers SC, Boffano P, Forouzanfar T. Consensus or controversy? the classification and treatment decision-making by 491 maxillofacial surgeons from around the world in three cases of a unilateral mandibular condyle fracture. Journal of Cranio-Maxillofacial Surgery. 2015;43(10):1952-60. | Excluded by abstract |
| 294 | Lezcano MF, Dias FJ, Chuhuaicura P, Navarro P, Fuentes R. Symmetry of mandibular movements: A 3D electromagnetic articulography technique applied on asymptomatic participants. Journal of Prosthetic Dentistry. 2020. | Excluded by abstract |
| 295 | Lloyd TE, Drage NA, Cronin AJ. The role of cone beam computed tomography in the management of unfavourable fractures following sagittal split mandibular osteotomy. Journal of Orthodontics. 2011;38(1):48-54. | Excluded by abstract |
| 296 | Lund K. Unusual fracture dislocation of the mandibular condyle in a 6-year-old child. International Journal of Oral Surgery. 1972;1(1):53-60. | Excluded by abstract |
| 297 | Meyer C, Serhir L, Boutemi P. Experimental evaluation of three osteosynthesis devices used for stabilizing condylar fractures of the mandible. Journal of Cranio-Maxillofacial Surgery. 2006;34(3):173-81. | Excluded by abstract |
| 298 | Nilesh K, Vande AV. Post-traumatic deformity of mandibular condyle: Descriptive review and proposal of treatment algorithm. Journal of Stomatology. 2020;73(1):36-43. | Excluded by abstract |
| 299 | Rozeboom AVJ, Dubois L, Lobbezoo F, Schreurs R, Milstein DMJ, de Lange J. Management of post-traumatic malocclusion: an alternative treatment. Oral Surgery. 2018;11(3):241-6. | Excluded by abstract |
| 300 | Sahm G, Witt E. Long-term results after childhood condylar fractures. A computer-tomographic study. European Journal of Orthodontics. 1989;11(2):154-60. | Excluded by abstract |
| 301 | Satake H, Yamada T, Kitamura N, Yoshimura T, Sasabe E, Yamamoto T. Post-surgical unilateral temporomandibular joint dislocation treated by open reduction followed by orthodontic treatment. International Journal of Oral and Maxillofacial Surgery. 2011;40(3):335-8. | Excluded by abstract |
| 302 | Scafati ST, Scafati CT. Versatility of anchor screws in craniofacial surgery. International Journal of Oral and Maxillofacial Surgery. 2011;40(2):227-8. | Excluded by abstract |
| 303 | Seeley-Hacker BL, Holmgren EP, Harper CW, Lauer CS, Van Citters DW. An Anatomic Predisposition to Mandibular Angle Fractures. Journal of Oral and Maxillofacial Surgery. 2020. | Excluded by abstract |
| 304 | Srivastava D, Mishra S, Jaetli V, Singh H. Bifid condyle secondary to traumatic condylar fracture. Journal of Oral and Maxillofacial Surgery, Medicine, and Pathology. 2014;26(4):501-5. | Excluded by abstract |
| 305 | Tabchouri N, Kadlub N, Diner PA, Picard A. Unusual costochondral bone graft complication. International Journal of Oral and Maxillofacial Surgery. 2013;42(11):1427-30. | Excluded by abstract |
| 306 | Tracy K, Gutta R. Are embrasure wires better than arch bars for intermaxillary fixation? Journal of Oral and Maxillofacial Surgery. 2015;73(1):117-22. | Excluded by abstract |
| 307 | Van Den Bergh B, Blankestijn J, Van Der Ploeg T, Tuinzing DB, Forouzanfar T. Conservative treatment of a mandibular condyle fracture: Comparing intermaxillary fixation with screws or arch bar. A randomised clinical trial. Journal of Cranio-Maxillofacial Surgery. 2015;43(5):671-6. | Excluded by abstract |
| 308 | Wang WH, Deng JY, Zhu J, Li M, Xia B, Xu B. Computer-assisted virtual technology in intracapsular condylar fracture with two resorbable long-screws. British Journal of Oral and Maxillofacial Surgery. 2013;51(2):138-43. | Excluded by abstract |
| 309 | Zrounba H, Lutz JC, Zink S, Wilk A. Epidemiology and treatment outcome of surgically treated mandibular condyle fractures. A five years retrospective study. Journal of Cranio-Maxillofacial Surgery. 2014;42(6):879-84. | Excluded by abstract |
| 310 | Becking AG, Zijderveld SA, Tuinzing DB. Management of posttraumatic malocclusion caused by condylar process fractures. Journal of Oral and Maxillofacial Surgery. 1998;56(12):1370-4. | Excluded by abstract |
| 311 | Chen I, Chang C-M, Chen MY-C, Chen K-J. Traumatic dislocation of the mandibular condyle into the middle cranial fossa treated by an intraoral approach. Journal of the Formosan Medical Association. 2019;118(7):1161-5. | Excluded by abstract |
| 312 | Chen LJ, Zhao MC, Pan XF, Wei YQ, Wang DY. X-Cephalometric Study of Different Parts of the Upper Airway Space and Changes in Hyoid Position following Mandibular Fractures. West Indian Medical Journal. 2013;62(7):642-8. | Excluded by abstract |
| 313 | Deffez JP, Themar P, Allain P, Berrada K, Bordais P, Brethaux J, et al. Dynamic Blockade in Cases of Fracture of the Condyle of Mandible and Immediate Post-Operative Developments Consecutive to Temporomandibular Ankylosis of the Child. Revue de Stomatologie et de Chirurgie Maxillo-Faciale. 1991;92(2):65-70. | Excluded by abstract |
| 314 | Douglas CR, Villaca Avoglio JL, de Oliveira H. Stomatognathic adaptive motor syndrome is the correct diagnosis for temporomandibular disorders. Medical Hypotheses. 2010;74(4):710-8. | Excluded by abstract |
| 315 | Escott EJ, Branstetter BF. Incidence and characterization of unifocal mandible fractures on CT. American Journal of Neuroradiology. 2008;29(5):890-4. | Excluded by abstract |
| 316 | Farronato G, Giannini L, Galbiati G, Stabilini SA, Sarcina M, Maspero C. Functional evaluation in orthodontic surgical treatment: long-term stability and predictability. Progress in Orthodontics. 2015;16. | Excluded by abstract |
| 317 | Frake PC, inventorIntramedullary mandibular condyle implants and method for application of the same patent US 08357162. 2013 Jan 22 2013. | Excluded by abstract |
| 318 | Ganeval A, Zink S, Del Pin D, Lutz JC, Wilk A, Barriere P. Modified Risdon approach for non-traumatic ramus surgery. Revue De Stomatologie Et De Chirurgie Maxillo-Faciale. 2012;113(2):96-9. | Excluded by abstract |
| 319 | Goormans F, Sun Y, Bila M, Schoenaers J, Geusens J, Luebbers H-T, et al. Accuracy of computer-assisted mandibular reconstructions with free fibula flap: Results of a single-center series. Oral Oncology. 2019;97:69-75. | Excluded by abstract |
| 320 | Hammer B, Schier P, Prein J. Osteosynthesis of condylar neck fractures: a review of 30 patients. British Journal of Oral & Maxillofacial Surgery. 1997;35(4):288-91. | Excluded by abstract |
| 321 | Handa Y. Modeling and Remodeling on Growing Condylar Process of the Mandible Histomorphometric Study under 18-Millimeter Undecalcified Ground Section. Acta Scholae Medicinalis Universitatis in Gifu. 1984;32(3):504-22. | Excluded by abstract |
| 322 | Hillerup S. Internal fixation of severely displaced mandibular condylar neck fracture with the aid of ramus osteotomy - A revised technique. International Journal of Oral and Maxillofacial Surgery. 1997;26(4):272-4. | Excluded by abstract |
| 323 | Hu Y, Zhang L, He D, Yang C, Chen M, Zhang S, et al. Simultaneous treatment of temporomandibular joint ankylosis with severe mandibular deficiency by standard TMJ prosthesis. Scientific Reports. 2017;7. | Excluded by abstract |
| 324 | Hyde N, Manisali M, Aghabeigi B, Sneddon K, Newman L. The role of open reduction and internal fixation in unilateral fractures of the mandibular condyle: a prospective study. British Journal of Oral & Maxillofacial Surgery. 2002;40(1):19-22. | Excluded by abstract |
| 325 | Ivashchenko NI. [Experience in using the surgical-orthodontic method proposed by V. V. Donskoi for treating mandibular fractures]. Stomatologiia. 1990;69(6):40-2. | Excluded by abstract |
| 326 | Kahl-Nieke B, Fischbach R. Effect of early orthopedic intervention on hemifacial microsomia patients: An approach to a cooperative evaluation of treatment results. American Journal of Orthodontics and Dentofacial Orthopedics. 1998;114(5):538-50. | Excluded by abstract |
| 327 | Kondoh T, Dolwick MF, Hamada Y, Seto K. Visually guided irrigation for patients with symptomatic internal derangement of the temporomandibular joint: A preliminary report. Oral Surgery Oral Medicine Oral Pathology Oral Radiology and Endodontics. 2003;95(5):544-51. | Excluded by abstract |
| 328 | Kuang S-J, He Y-Q, Zheng Y-H, Zhang Z-G. Open reduction and internal fixation of mandibular condylar fractures A national inpatient sample analysis, 2005-2014. Medicine. 2019;98(37). | Excluded by abstract |
| 329 | Kubein D, Luhr HG, Jaeger A, Schauer HW, Von Ehrlich V. Diagnosis of the Relationship of the Mandibular Joint to Occlusion Intraoperative Control Technique for the Optimization of Orthodontic-Surgical Interventions in Reference to Plate Osteosynthesis. Fortschritte der Kieferorthopaedie. 1987;48(4):267-75. | Excluded by abstract |
| 330 | Laemmle M, Jung B, Voss P, Schmelzeisen R. Treatment of Subcondylar and Condylar Fractures: Possibilities and Chances of Success. Informationen Aus Orthodontie Und Kieferorthopaedie. 2017;49(3):198-201. | Excluded by abstract |
| 331 | Lauer G, Haim D, Proff P, Richter G, Pradel W, Fanghaenel J, et al. Plate osteosynthesis of the mandibular condyle. Annals of Anatomy-Anatomischer Anzeiger. 2007;189(4):412-7. | Excluded by abstract |
| 332 | Liu M-Q, Chen H-M, Yap AUJ, Fu K-Y. Condylar remodeling accompanying splint therapy: a cone-beam computerized tomography study of patients with temporomandibular joint disk displacement. Oral Surgery Oral Medicine Oral Pathology Oral Radiology. 2012;114(2):259-65. | Excluded by abstract |
| 333 | Marker P, Nielsen A, Bastian HL. Fractures of the mandibular condyle. Part 1: Patterns of distribution of types and causes of fractures in 348 patients. British Journal of Oral & Maxillofacial Surgery. 2000;38(5):417-21. | Excluded by abstract |
| 334 | McGrath CJR, Egbert MA, Tong DC, Myall RWT. Unusual presentations of injuries associated with the mandibular condyle in children. British Journal of Oral & Maxillofacial Surgery. 1996;34(4):311-4. | Excluded by abstract |
| 335 | Mejia-Gomez CM, Ramirez-Yanez GO. Mandibular Condylar Aplasia Treated with a Functional Approach. Journal of Clinical Pediatric Dentistry. 2013;38(2):179-84. | Excluded by abstract |
| 336 | Miloro M. Endoscopic-assisted repair of subcondylar fractures. Oral Surgery Oral Medicine Oral Pathology Oral Radiology and Endodontics. 2003;96(4):387-91. | Excluded by abstract |
| 337 | Minervini G, Lucchese A, Perillo L, Serpico R, Minervini G. Unilateral superior condylar neck fracture with dislocation in a child treated with an acrylic splint in the upper arch for functional repositioning of the mandible. Cranio-the Journal of Craniomandibular & Sleep Practice. 2017;35(5):337-41. | Excluded by abstract |
| 338 | Napolitano G, Sodano A, Califano L, Grassi R, Brunese L. Multidetector Row Computed Tomography with Multiplanar and 3D Images in the Evaluation of Posttreatment Mandibular Fractures. Seminars in Ultrasound Ct and Mri. 2009;30(3):181-7. | Excluded by abstract |
| 339 | Panula K, Finne K, Oikarinen K. Incidence of complications and problems related to orthognathic surgery: A review of 655 patients. Journal of Oral and Maxillofacial Surgery. 2001;59(10):1128-36. | Excluded by abstract |
| 340 | Paoli JR, Fabie L, Dodart L, Lauwers F, Boutault F, Fabie M. Mandibular fractures in sportsmen: A retrospective study of 48 cases. Revue de Stomatologie et de Chirurgie Maxillo-Faciale. 1999;100(6):306-10. | Excluded by abstract |
| 341 | Park C-H, Chung KJ, Kim TG, Lee JH, Kim I-K, Kim Y-H. Big Data Statistical Analysis of Facial Fractures in Korea. Journal of Korean Medical Science. 2020;35(7). | Excluded by abstract |
| 342 | Patel R, Reid RR, Poon CS. Multidetector Computed Tomography of Maxillofacial Fractures: The Key to High-Impact Radiological Reporting. Seminars in Ultrasound Ct and Mri. 2012;33(5):410-7. | Excluded by abstract |
| 343 | Rastogi S, Sharma S, Kumar S, Reddy MP, Indra BN. Fracture of mandibular condyle-to open or not to open: an attempt to settle the controversy. Oral Surgery Oral Medicine Oral Pathology Oral Radiology. 2015;119(6):608-13. | Excluded by abstract |
| 344 | Romeo A, Pinto A, Cappabianca S, Scaglione M, Brunese L. Role of Multidetector Row Computed Tomography in the Management of Mandible Traumatic Lesions. Seminars in Ultrasound Ct and Mri. 2009;30(3):174-80. | Excluded by abstract |
| 345 | Sala-Perez S, Vazquez-Delgado E, Rodriguez-Baeza A, Gay-Escoda C. Bifid mandibular condyle A disorder in its own right? Journal of the American Dental Association. 2010;141(9):1076-85. | Excluded by abstract |
| 346 | Schoning H, Emshoff R. Primary temporary AO plate reconstruction of the mandible. Oral Surgery Oral Medicine Oral Pathology Oral Radiology and Endodontology. 1998;86(6):667-72. | Excluded by abstract |
| 347 | Sharma AP, Hondorp B, Gaiduchik A, Baba NZ, Thakker J, Inman JC. Management of Malocclusion after Maxillofacial Trauma. Facial Plastic Surgery. 2017;33(6):562-70. | Excluded by abstract |
| 348 | Tanaka E, Detamore MS, Tanimoto K, Kawai N. Lubrication of the temporomandibular joint. Annals of Biomedical Engineering. 2008;36(1):14-29. | Excluded by abstract |
| 349 | Thoren H, Iizuka T, Hallikainen D, Lindqvist C. Radiologic changes of the temporomandibular joint after condylar fractures in childhood. Oral Surgery Oral Medicine Oral Pathology Oral Radiology and Endodontics. 1998;86(6):738-45. | Excluded by abstract |
| 350 | Thoren H, Iizuka T, Hallikainen D, Nurminen M, Lindqvist C. An epidemiological study of patterns of condylar fractures in children. British Journal of Oral & Maxillofacial Surgery. 1997;35(5):306-11. | Excluded by abstract |
| 351 | Tullio A, Sesenna E. Role of surgical reduction of condylar fractures in the management of panfacial fractures. British Journal of Oral & Maxillofacial Surgery. 2000;38(5):472-6. | Excluded by abstract |
| 352 | Uematsu H, Ichida T, Masumi S, Morimoto Y, Tanaka T, Konoo T, et al. Diagnostic image analyses of activator treated temporomandibular joint in growth and maturing stages. Cranio-the Journal of Craniomandibular & Sleep Practice. 2002;20(4):254-63. | Excluded by abstract |
| 353 | Ugolini A, Mapelli A, Segu M, Galante D, Sidequersky FV, Sforza C. Kinematic analysis of mandibular motion before and after orthognathic surgery for skeletal Class III malocclusion: A pilot study. Cranio-the Journal of Craniomandibular & Sleep Practice. 2017;35(2):94-100. | Excluded by abstract |
| 354 | Wagner F, Strasz M, Traxler H, Schicho K, Seemann R. Evaluation of an experimental oblique plate for osteosynthesis of mandibular condyle fractures. Oral Surgery Oral Medicine Oral Pathology Oral Radiology. 2017;124(6):537-41. | Excluded by abstract |
| 355 | Wang P, Yang J, Yu Q. MR Imaging Assessment of Temporomandibular Joint Soft Tissue Injuries in Dislocated and Nondislocated Mandibular Condylar Fractures. American Journal of Neuroradiology. 2009;30(1):59-63. | Excluded by abstract |
| 356 | Wang Y, Chen Y, Liu Y. Innovative Application of Orthodontic Treatment in Jaw Fractures. Journal of China Medical University. 2011;40(7):619-21,48. | Excluded by abstract |
| 357 | Wilson AW, Ethunandan M, Brennan PA. Transmasseteric antero-parotid approach for open reduction and internal fixation of condylar fractures. British Journal of Oral & Maxillofacial Surgery. 2005;43(1):57-60. | Excluded by abstract |
| 358 | Yoshii T, Hamamoto Y, Muraoka S, Teranobu O, Shigeta Y, Komori T. Traumatic dislocation of the mandibular condyle into the temporal fossa in a child. Journal of Trauma-Injury Infection and Critical Care. 2000;49(4):764-6. | Excluded by abstract |
| 359 | Zhang S, Wu J, Xu B, Shi J, Shen SGF, Gui H. Features and management of intracranial mandibular condyle dislocation after trauma. Cranio-the Journal of Craniomandibular & Sleep Practice. 2014;32(1):63-7. | Excluded by abstract |
| 360 | Zhou H-H, Lv K, Yang R-T, Li Z, Li Z-B. Risk factor analysis and idiographic features of mandibular coronoid fractures: A retrospective case-control study. Scientific Reports. 2017;7. | Excluded by abstract |
| 361 | Zhou H-H, Lv K, Yang R-T, Li Z, Yang X-W, Li Z-B. Clinical, retrospective case-control study on the mechanics of obstacle in mouth opening and malocclusion in patients with maxillofacial fractures. Scientific Reports. 2018;8. | Excluded by abstract |
| 362 | Zhou Z, Li Za, Ren J, He M, Huang Y, Tian W, et al. Digital diagnosis and treatment of mandibular condylar fractures based on Extensible Neuro imaging Archive Toolkit (XNAT). Plos One. 2018;13(2). | Excluded by abstract |
| 363 | Greer B, Alpert B. The challenge of determining correct occlusion in complex mandibular fractures. Journal of Oral and Maxillofacial Surgery. 2011;69(9):e-88. | Excluded by abstract |
| 364 | Ito Y, Sumiya N, Kato Y, Hayakawa O, Akizuki A, Kameyama K. Use of acutrac bone screws in fractures of the mandibular condyle. Japanese Journal of Plastic and Reconstructive Surgery. 2005;48(3):291-8. | Excluded by abstract |
| 365 | Jung GS, Kim TK, Lee JW, Yang JD, Chung HY, Cho BC, et al. The effect of a condylar repositioning plate on condylar position and relapse in two-jaw surgery. Archives of Plastic Surgery. 2017;44(1):19-25. | Excluded by abstract |
| 366 | Kannadasan K, Shenoy K V, Kengagsubbiah S, Sathyabhama V, Priya V. Extra corporeal fixation of fractured mandibular condyle. Journal of Clinical and Diagnostic Research. 2014;8(9):ZD41-ZD3. | Excluded by abstract |
| 367 | Defabianis P. TMJ fractures in children and adolescents: treatment guidelines. J Clin Pediatr Dent. 2003;27(3):191-9. | Excluded by abstract |
| 368 | Khan AQ, Khan MS, Sherwani MKA, Agarwal R. Role of valgus osteotomy and fixation with dynamic hip screw and 120° double angle barrel plate in the management of neglected and ununited femoral neck fracture in young patients. Journal of Orthopaedics and Traumatology. 2009;10(2):71-8. | Excluded by abstract |
| 369 | Khan I, Sybil D, Kaur M, Iftikhar I, Manzoor N, Khan R, et al. Novel Aberrant Mandibular Angle Foramen: A Novel Aberrancy mimicking mandibular angle fracture. Oral and Maxillofacial Surgery Cases. 2020;6(3). | Excluded by abstract |
| 370 | Krzemień J, Bańczyk Ł, Baron S, Niedzielska I. The influence of the condylar fracture treatment method on mandible dynamics. Dental and Medical Problems. 2017;54(4):353-9. | Excluded by abstract |
| 371 | Li ZX, Chen CJ. Condylar resorption: Etiology manifestations and modern restorative treatment. Chinese Journal of Tissue Engineering Research. 2014;18(46):7482-6. | Excluded by abstract |
| 372 | Murad R, Silva CEXSR, Rodriguez AC, Costa DM, Oliveira V, Martins M, et al. Closed treatment for condylar process fracture, is the best option? International Journal of Oral and Maxillofacial Surgery. 2019;48:200. | Excluded by abstract |
| 373 | Pinto CMSDA, Araújo PM, Lima FN, Osterne RLV, Santana E, Nogueira RLM. Lower face asymmetry treated by orthognathic surgery in two surgical times. International Journal of Oral and Maxillofacial Surgery. 2011;40(10):1199. | Excluded by abstract |
| 374 | Rock A, Hudson C, Vazquez D, Lazow S. Evaluation of the inion resorbable plating system for open reduction internal fixation (ORIF) of mandible fractures. Journal of Oral and Maxillofacial Surgery. 2011;69(9):e-90. | Excluded by abstract |
| 375 | Silveira RL, Ranuzia I, Melo MFS, De Oliveira RA, De Brito AA, Vidigal VL. Traumatic Anterosuperior Dislocation of the Intact Mandibular Condyle into the Temporal Fossa. Craniomaxillofacial Trauma and Reconstruction. 2018;11(4):296-301. | Excluded by abstract |
| 376 | Thompson J, Malandris M, Anderson P. A pitfall in the radiological diagnosis of paediatric mandibular condylar fractures. Asian Journal of Oral and Maxillofacial Surgery. 2007;19(1):54-7. | Excluded by abstract |
| 377 | Wiktor-Stoma A, Rahnama M, Bozyk J, Borowicz J. The treatment of the condylar fracture of the mandible and the complications using conservative-orthopedic treatment. Annales Universitatis Mariae Curie-Sklodowska, Sectio DDD: Pharmacia. 2011;24(2):91-6. | Excluded by abstract |
| 378 | Yildirim Y, Keller EE. Remodeling of displaced condylar fractures with functional treatment: High-quality radiographic documentation in three-patient series. Craniomaxillofacial Trauma and Reconstruction. 2014;8(4):334-40. | Excluded by abstract |
| 379 | Yu H, Shen S. Virtual surgical planning in the treatment of bony facial asymmetry. International Journal of Computer Assisted Radiology and Surgery. 2018;13:S65-S6. | Excluded by abstract |
| 380 | Biondi AM, Cortese SG, Sarmiento Z. Asimetría facial en paciente preescolar. Bol Asoc Argent Odontol Niños. 2005;34(3):8-11. | Excluded by abstract |
| 381 | Eid RMR, Takaoka LAMV, Matos MF, Lavoura MG, Siqueira JTTd. Fratura bicondilar em criança: tratamento conservador com aparelho ortopédico: acompanhamento longitudinal de 4 anos: parte 2. J bras ortodontia ortop maxilar. 1998;3(13):31-3. | Excluded by abstract |
| 382 | Maniglia JV, Molina FD, Marques CG, Carvalho TBOd, Pinheiro LFA. Fratura do processo condilar da mandíbula. Arq ciênc saúde. 2009;16(3):137-40. | Excluded by abstract |
| 383 | Marcolino PRB, Gomes RSdS, Silva PAd. Fratura de Côndilo Mandibular em Criança: Tratamento Ortopédico-Funcional. Rev cir traumatol buco-maxilo-fac. 2014;14(2):43-8. | Excluded by abstract |
| 384 | Maricevich P, Pantoja E, Mansur A, Peixoto A, Amando J, Borges PYV, et al. Prototipagem: aplicações na cirurgia crâniomaxilo- facial do Instituto Nacional de Traumatologia e Ortopedia (INTO)- RJ. Rev bras cir plást. 2015;30(4):626-32. | Excluded by abstract |
| 385 | Ostrosky A, Mikler A, Févola L, Parisi G, Virgillito A, Suárez F. Osteosíntesis rígida interna en fracturas mandibulares: ¿una o dos placas de fijación? Salud bucal. 2001(89):18-20. | Excluded by abstract |
| 386 | Rosé MM. Fractura y remodelación condílea. Ortodoncia. 2002;66(131):50-8. | Excluded by abstract |
| 387 | Rossinol VL, Souza KAd, Pinto MVdM, Júnior PBdS, Prates LS. Fratura bicondilar da articulação temporomandibular. Fisioter Bras. 2006;7(4):317-21. | Excluded by abstract |
| 388 | Siqueira JTTd. Fratura bicondilar em crianças: tratamento conservador com aparelho ortopédico. J bras ortodontia ortop maxilar. 1997;2(9):19-34. | Excluded by abstract |
| 389 | Albanese S, Caruso F, di Lauro F, Bucci E. [Orthopedic treatment of mandibular condylar fractures]. Arch Stomatol (Napoli). 1982;23(1):213-27. | Excluded; missing fulltext |
| 390 | Berthouze E, Sagne D, Momege B, Achard R. [Treatment of mandibular fractures in children. Our therapeutic approach (author's transl)]. Rev Stomatol Chir Maxillofac. 1980;81(5):285-8. | Excluded; missing fulltext |
| 391 | Budai M. [Treatment of the fracture of the caput and collum of the mandible using the Andresen-Häupl appliance]. Fogorv Sz. 1989;82(9):277-9. | Excluded; missing fulltext |
| 392 | Cadenat E, Cadenat H, Joniot B, Gely, Blank P. [Long-term results of condylar fractures of the lower jaw treated by orthopedic methods]. Toulouse Med. 1962;63:1022-3. | Excluded; missing fulltext |
| 393 | Cadenat H, Boutault F, Comiti G, Combelles R, Fabie M, Baro JP. [Value of the surgical treatment of subcondylar fractures. Apropos of a comparative study of 24 cases of luxation-fractures treated by to-and-fro osteosynthesis or by active protrusive mobilization]. Rev Stomatol Chir Maxillofac. 1984;85(6):455-64. | Excluded; missing fulltext |
| 394 | Catapano B, Curci G. [Follow-up studies on cases of condylar fracture treated by orthodontic means]. Rass Int Stomatol Prat. 1966;17(2):119-27. | Excluded; missing fulltext |
| 395 | Cerrada M L, Gonzáles R N, Marrone M L, Ceballos G. Tratamiento de las fracturas de cóndilo mandibular en la IAHULA: 1999-2004. Rev venez cir ortop traumatol. 2007;39(2):47-52. | Excluded; missing fulltext |
| 396 | Cuéllar J, Santana J, Núñez C, Villanueva J. Surgical or conservative treatment for mandibular condyle fractures. Medwave. 2018;18(7):e7352. | Excluded; missing fulltext |
| 397 | De Michelis B, Odasso M. [Long term results in fractures of the mandibular condyle treated with orthopedic therapy]. Minerva Stomatol. 1967;16(1):1-11. | Excluded; missing fulltext |
| 398 | Gerlach KL, Kahl B, Berg S. [The treatment of condylar fractures in children]. Dtsch Zahnarztl Z. 1991;46(1):43-5. | Excluded; missing fulltext |
| 399 | Iannetti G, Martucci E, Chimenti C, Pelo S. [Treatment of mandibular condyle fractures]. Minerva Stomatol. 1984;33(1):165-71. | Excluded; missing fulltext |
| 400 | Iannetti G, Martucci E, Pelo S, Peroni S. [Therapy of monocondylar fractures in growing children]. Minerva Stomatol. 1984;33(3):527-32. | Excluded; missing fulltext |
| 401 | Ioannidis GP, Kim AE. [Late results of the treatment of fractures of the mandibular condyle]. Stomatologiia (Mosk). 1978;57(1):52-4. | Excluded; missing fulltext |
| 402 | Kadlub N, Trost O, Duvernay A, Parmentier J, Wirth C, Malka G. [Orthopaedic treatment of extraarticular condylar fractures of the mandible: retrospective study of 39 unilateral cases]. Rev Stomatol Chir Maxillofac. 2008;109(5):301-5; discussion 5-6. | Excluded; missing fulltext |
| 403 | Karłowska I, Wilk G, Wedrychowska-Szulc B. [Radiological and clinical evaluation of the late results of the functional-orthopedic treatment of subcondylar fractures with displacement of the mandibular capitulum]. Czas Stomatol. 1986;39(12):830-6. | Excluded; missing fulltext |
| 404 | Klewansky P, Saad M. [Anatomic clinical studies of fractures of the mandibular condyles]. Rev Fr Odontostomatol. 1968;15(8):1057-86. | Excluded; missing fulltext |
| 405 | Lachard J, Zattara H, Romette JM, Vitton J. Indications for osteosynthesis in the treatment of low sub-condylar fractures. Revue de Stomatologie et de Chirurgie Maxillo-Faciale. 1971;72(2):201-4. | Excluded; missing fulltext |
| 406 | Liu CK, Xu J, Tan XY, Liu SX, Hu KJ, Hu M. [Comparative efficacies of non-surgical treatment for pediatric and adult sagittal fractures of mandibular condyle]. Zhonghua Yi Xue Za Zhi. 2013;93(32):2578-80. | Excluded; missing fulltext |
| 407 | Marinelli M, Agrestini F, Badia DM, Vallogini P. [Functional therapy of condylar fractures]. Minerva Stomatol. 1985;34(2):399-404. | Excluded; missing fulltext |
| 408 | Melkiĭ VI. [Distraction method of treating mandibular condylar fractures]. Stomatologiia (Mosk). 1981;60(5):51-3. | Excluded; missing fulltext |
| 409 | Mercier J, Huet P, Perrin JP. Functional management of fractures of the mandibular condyle. Revue de Stomatologie et de Chirurgie Maxillo-Faciale. 2000;101(4):203-6. | Excluded; missing fulltext |
| 410 | Mercier J, Lemoine V, Gaillard A, Delaire J. [Results of treatment of mandibular fractures in 27 children (author's transl)]. Rev Stomatol Chir Maxillofac. 1980;81(5):296-300. | Excluded; missing fulltext |
| 411 | Brandt N, Knak G. [The effect of fracture of the condylar process of the mandible on the function and the morphology of the mandible in children and adolescents]. Dtsch Stomatol. 1969;19(4):241-8. | Excluded; missing fulltext |
| 412 | Crivello Junior O. Estudo comparativo dos tratamentos ortopédicos nas fraturas do côndilo mandibular. 1995. p. 70-. | Excluded; missing fulltext |
| 413 | Pogorzelska-Stronczak B, Stanecka M. [Late results of conservative orthopaedic treatment of fractures of the mandibular condyle in children]. Czas Stomatol. 1970;23(8):969-74. | Excluded; missing fulltext |
| 414 | Prayer Galletti S, Marchesi A, Cudia G. [The functional therapy of condylar fractures: a kinesiographic follow-up]. Minerva Stomatol. 1990;39(10):849-53. | Excluded; missing fulltext |
| 415 | Quan XY, Qin M, Kang YF, Zhang Y, Zhao YM. [Radiographic and masticatory physiologic evaluation after conservative treatment of condylar fractures in children and adolescents]. Zhonghua Kou Qiang Yi Xue Za Zhi. 2016;51(1):30-5. | Excluded; missing fulltext |
| 416 | Rikhotso E, Ferretti C. A prospective audit over a six month period of condylar fractures at Chris Hani Baragwanath Hospital. Sadj. 2008;63(4):222-5, 8-9. | Excluded; missing fulltext |
| 417 | Rosignoli M, Paludetti G, D'Alatri L, Maurizi M. [Our experience with mandibular fractures]. Ann Ital Chir. 1990;61(3):255-63. | Excluded; missing fulltext |
| 418 | Röthler G, Strobl H, Strobl V, Norer B, Waldhart E. [Fractures of the mandibular collum in childhood--a long-term follow-up with orthopantomography]. Fortschr Kiefer Gesichtschir. 1996;41:146-7. | Excluded; missing fulltext |
| 419 | Schendel KU, Wiesinger A, Gademann G, Komposch G. [Results of functional activator treatment of collum fractures in the growth period]. Dtsch Zahnarztl Z. 1991;46(11):726-8. | Excluded; missing fulltext |
| 420 | Schiavoni R, Cascone P, Silvestri A, Chimenti C. [Use of bionator in the treatment of monocondylar fractures in young patients]. Mondo Ortod. 1981;6(1):36-44. | Excluded; missing fulltext |
| 421 | Schienbein H. [Treatment of fractures of the temporomandibular joint with an activator (I)]. Quintessenz. 1976;27(1):21-7. | Excluded; missing fulltext |
| 422 | Schienbein H. [Treatment of mandibular joint fractures with an activator (II)]. Quintessenz. 1976;27(2):41-50. | Excluded; missing fulltext |
| 423 | Schienbein H. [Treatment of mandibular joint fractures with an activator (III)]. Quintessenz. 1976;27(3):31-7. | Excluded; missing fulltext |
| 424 | Schienbein H. [Treatment of temporo-mandibular joint fractures with an activator (V)]. Quintessenz. 1976;27(5):31-7. | Excluded; missing fulltext |
| 425 | Schienbein H. [Treatment of temporomandibular joint fractures with the activator IV]. Quintessenz. 1976;27(4):27-32. | Excluded; missing fulltext |
| 426 | Schienbein H. Treatment of temporomandibular fractures with an activator (II). Quintessence Int Dent Dig. 1977;8(7):9-18. | Excluded; missing fulltext |
| 427 | Silvestri A, Accivile E. [Functional therapy in the treatment of fractures of the mandibular condyle]. Mondo Ortod. 1986;11(2):45-51. | Excluded; missing fulltext |
| 428 | Sonnenburg M, Sonnenburg I, Herbst B, Wendorf B. [Maxillofacial fractures in children]. Stomatol DDR. 1977;27(3):186-92. | Excluded; missing fulltext |
| 429 | Spitzer WJ, Hirschfelder U, Müssig D, Hertrich K. [Findings following functional orthopedic treatment of TMJ fractures in the growth period]. Dtsch Zahnarztl Z. 1991;46(1):57-9. | Excluded; missing fulltext |
| 430 | Spitzer WJ, Zschiesche S. [Results of functional orthodontic treatment of mandibular condyle fractures during growth]. Dtsch Zahnarztl Z. 1986;41(2):174-8. | Excluded; missing fulltext |
| 431 | Sysoliatin PG, Zheleznyĭ PA, Ishchenko NA. [The results of the surgical treatment of fractures of the mandibular condyle in children]. Stomatologiia (Mosk). 1992(3-6):45-8. | Excluded; missing fulltext |
| 432 | Tay AG, Yeow VK, Tan BK, Sng K, Huang MH, Foo CL. A review of mandibular fractures in a craniomaxillofacial trauma centre. Ann Acad Med Singap. 1999;28(5):630-3. | Excluded; missing fulltext |
| 433 | Ferrara S, Piombino P, Sbordone C, Spinzia A, Califano L. Recovery of mandibular functionality after closed treatment of mandibular condylar fractures with a modified bionator of balters. European Surgical Research. 2010;45(3-4):288-9. | Excluded; missing fulltext |
| 434 | van Hove A, Lolom P, Sapanet M, Descrozailles JM. [Mandibular fractures. Retrospective study of the experience of the Department of Maxillo-facial Surgery and Stomatology of the University of Poitiers Medical Center from 1978 to 1997]. Rev Stomatol Chir Maxillofac. 2000;101(6):309-18. | Excluded; missing fulltext |
| 435 | Weiskopf J. [The fractures of the mandibular ramus]. Dtsch Stomatol. 1967;17(1):3-20. | Excluded; missing fulltext |
| 436 | Wernicke M. [Contributions to the surgical treatment of fractures of the temporomandibular joint]. Dtsch Stomatol. 1968;18(10):733-40. | Excluded; missing fulltext |
| 437 | Zarzar Castro E, Sancho P MA. Tratamiento ortopédico funcional de las fracturas de cóndilo mandibular. Rev chil ortod. 1984;1(2):97-106. | Excluded; missing fulltext |
| 438 | Cabral LC, Alves GM, Furtado LM, Fernandes Neto AJ, Simamoto Junior PC. Changes in mandibular and articular dynamics associated with surgical versus nonsurgical treatment of mandibular condylar fractures: a systematic review with meta-analysis. Oral Surgery Oral Medicine Oral Pathology Oral Radiology. 2020;129(4):311-21. | Excluded; not relevant |
| 439 | Carlsen A, Marcussen M. Spontaneous fractures of the mandible concept & treatment strategy. Medicina Oral, Patologia Oral y Cirugia Bucal. 2016;21(1):e88-e94. | Excluded; not relevant |
| 440 | Fiorelli G, Merlo P, Dalstra M, Melsen B. Reposicionamento mandibular em pacientes adultos ­ uma alternativa à cirurgia? Um acompanhamento de dois anos. Ortho Sci, Orthod sci pract. 2019;12(46):46-59. | Excluded; not relevant |
| 441 | Ghazal G, Jaquiéry C, Hammer B. Non-surgical treatment of mandibular fractures--survey of 28 patients. Int J Oral Maxillofac Surg. 2004;33(2):141-5. | Excluded; not relevant |
| 442 | Griffiths H, Townend J. Anesthesia of the inferior alveolar and lingual nerves as a complication of a fractured condylar process. J Oral Maxillofac Surg. 1999;57(1):77-9. | Excluded; not relevant |
| 443 | Han L, Long T, Tang W, Liu L, Jing W, Tian W-D, et al. Correlation between Condylar Fracture Pattern after Parasymphyseal Impact and Condyle Morphological Features: A Retrospective Analysis of 107 Chinese Patients. Chinese Medical Journal. 2017;130(4):420-7. | Excluded; not relevant |
| 444 | Hou J, Chen L, Wang T, Jing W, Tang W, Long J, et al. A new surgical approach to treat medial or low condylar fractures: the minor parotid anterior approach. Oral Surgery Oral Medicine Oral Pathology Oral Radiology. 2014;117(3):283-8. | Excluded; not relevant |
| 445 | Ingole PD, Garg A, Shenoi SR, Badjate SJ, Budhraja N. Comparison of intermaxillary fixation screw versus eyelet interdental wiring for intermaxillary fixation in minimally displaced mandibular fracture: A randomized clinical study. Journal of Oral and Maxillofacial Surgery. 2014;72(5):958.e1-.e7. | Excluded; not relevant |
| 446 | Kaul RP, Sagar S, Singhal M, Kumar A, Jaipuria J, Misra M. Burden of maxillofacial trauma at level 1 trauma center. Craniomaxillofacial Trauma and Reconstruction. 2014;7(2):126-30. | Excluded; not relevant |
| 447 | Laster Z, Muska EA, Nagler R. Pediatric mandibular fractures: Introduction of a novel therapeutic modality. Journal of Trauma-Injury Infection and Critical Care. 2008;64(1):225-9. | Excluded; not relevant |
| 448 | Laurentjoye M, Majoufre-Lefebvre C, Caix P, Siberchicot F, Ricard AS. Treatment of mandibular fractures with Michelet technique: manual fracture reduction without arch bars. J Oral Maxillofac Surg. 2009;67(11):2374-9. | Excluded; not relevant |
| 449 | Li Z, Li ZB, Shang ZJ, Wu ZX. An unusual type of superolateral dislocation of mandibular condyle: discussion of the causative mechanisms and clinical characteristics. J Oral Maxillofac Surg. 2009;67(2):431-5. | Excluded; not relevant |
| 450 | Li Z, Zhang W, Li ZB, Li JR. Abnormal union of mandibular fractures: a review of 84 cases. J Oral Maxillofac Surg. 2006;64(8):1225-31. | Excluded; not relevant |
| 451 | Lin F-Y, Wu C-I, Cheng H-T. Mandibular Fracture Patterns at a Medical Center in Central Taiwan: A 3-Year Epidemiological Review. Medicine. 2017;96(51). | Excluded; not relevant |
| 452 | Loong CL, Razak NHA, Pohchi A, Alam MK. Mandibular condylar fracture at hospital universiti sains Malaysia (HUSM): A five year restrospective study. International Medical Journal. 2013;20(4):483-4. | Excluded; not relevant |
| 453 | McLeod NM, Saeed NR. Treatment of fractures of the mandibular condylar head with ultrasound-activated resorbable pins: early clinical experience. Br J Oral Maxillofac Surg. 2016;54(8):872-7. | Excluded; not relevant |
| 454 | Nikkerdar N, Azizi B, Golshah A, Asadi M. Maxillofacial fractures in patients treated at two hospitals of Kermanshah City, Iran. Indian Journal of Public Health Research and Development. 2017;8(2):374-9. | Excluded; not relevant |
| 455 | Noda K, Hosaka Y, Muramatsu H, Ueda T. A ten-year statistical study facial bone fractures at Showa University Hospital. Journal of the Showa Medical Association. 2005;65(4):325-36. | Excluded; not relevant |
| 456 | Nogami S, Yamauchi K, Bottini GB, Morishima H, Sai Y, Otake Y, et al. Mandibular fractures and dental injuries sustained during baseball and softball over 14 years in a Japanese population: A retrospective multicentre study. Dent Traumatol. 2020;36(2):156-60. | Excluded; not relevant |
| 457 | Oikarinen K, Altonen M, Kauppi H, Laitakari K. Treatment of mandibular fractures. Need for rigid internal fixation. J Craniomaxillofac Surg. 1989;17(1):24-30. | Excluded; not relevant |
| 458 | Seemann R, Undt G, Lauer G, Holawe S, Schicho K, Czerny C, et al. Is failure of condylar neck osteosynthesis predictable based on orthopantomography? Oral Surgery Oral Medicine Oral Pathology Oral Radiology and Endodontology. 2011;111(3):362-71. | Excluded; not relevant |
| 459 | Shirley B, Morris C, Tiwana PS. The effect of active orthodontic treatment on mandibular fracture injury patterns. Journal of Oral and Maxillofacial Surgery. 2012;70(9):e105-e6. | Excluded; not relevant |
| 460 | Sikora M, Olszowski T, Sielski M, Stąpor A, Janiszewska-Olszowska J, Chlubek D. The use of the transparotid approach for surgical treatment of condylar fractures - Own experience. J Craniomaxillofac Surg. 2015;43(10):1961-5. | Excluded; not relevant |
| 461 | Sirimaharaj W, Pyungtanasup K. The epidemiology of mandibular fractures treated at Chiang Mai University Hospital: A review of 198 cases. Journal of the Medical Association of Thailand. 2008;91(6):868-74. | Excluded; not relevant |
| 462 | Terai H, Shimahara M. Closed treatment of condylar fractures by intermaxillary fixation with thermoforming plates. British Journal of Oral & Maxillofacial Surgery. 2004;42(1):61-3. | Excluded; not relevant |
| 463 | Utley DS, Utley JD, Koch RJ, Goode RL. Direct bonded orthodontic brackets for maxillomandibular fixation. Laryngoscope. 1998;108(9):1338-45. | Excluded; not relevant |
| 464 | Vajgel A, Santos TDS, Camargo IB, De Oliveira DM, Laureano Filho JR, De Holanda Vasconcellos RJ, et al. Treatment of condylar fractures with an intraoral approach using an angulated screwdriver: Results of a multicentre study. Journal of Cranio-Maxillofacial Surgery. 2015;43(1):34-42. | Excluded; not relevant |
| 465 | van den Bergh B, de Mol van Otterloo JJ, van der Ploeg T, Tuinzing DB, Forouzanfar T. IMF-screws or arch bars as conservative treatment for mandibular condyle fractures: Quality of life aspects. J Craniomaxillofac Surg. 2015;43(7):1004-9. | Excluded; not relevant |
| 466 | Yano K, Nishikawa K, Sano T, Okano T. Relationship between appearance of a double contour on the mandibular condyle and the change in articular disc position after splint therapy. Oral Surgery Oral Medicine Oral Pathology Oral Radiology and Endodontology. 2009;108(4):E30-E4. | Excluded; not relevant |
| 467 | You HJ, Moon KC, Yoon ES, Lee BI, Park SH. Clinical and radiological outcomes of transoral endoscope-assisted treatment of mandibular condylar fractures. International Journal of Oral and Maxillofacial Surgery. 2016;45(3):284-91. | Excluded; not relevant |
| 468 | Zhang Q-B, Zhang Z-Q, Chen D, Zhao Y. Epidemiology of maxillofacial injury in children under 15 years of age in southern China. Oral Surgery Oral Medicine Oral Pathology Oral Radiology. 2013;115(4):436-41. | Excluded; not relevant |
| 469 | Bruckmoser E, Undt G. Management and outcome of condylar fractures in children and adolescents: A review of the literature. Oral Surgery Oral Medicine Oral Pathology Oral Radiology. 2012;114(5):S86-S106. | Excluded; not a clinical study |
| 470 | García-Guerrero I, Ramírez JM, Gómez de Diego R, Martínez-González JM, Poblador MS, Lancho JL. Complications in the treatment of mandibular condylar fractures: Surgical versus conservative treatment. Ann Anat. 2018;216:60-8. | Excluded; not a clinical study |
| 471 | Laine P, Kontio R, Salo A, Mesimäki K, Lindqvist C, Suuronen R. Secondary correction of malocclusion after treatment of maxillofacial trauma. J Oral Maxillofac Surg. 2004;62(10):1312-20. | Excluded; not a clinical study |
| 472 | Leake DL, Leake RD, Davee JS, Hansen RW. Definitive treatment of mandibular fractures in young children. Oral Surg Oral Med Oral Pathol. 1973;36(2):164-9. | Excluded; not a clinical study |
| 473 | Malkin M, Kresberg H, Mandel L. SUBMANDIBULAR APPROACH FOR OPEN REDUCTION OF CONDYLAR FRACTURE. Oral Surg Oral Med Oral Pathol. 1964;17:152-7. | Excluded; not a clinical study |
| 474 | Maron G, Kuhmichel A, Schreiber G. Secondary Treatment of Malocclusion/Malunion Secondary to Condylar Fractures. Atlas Oral Maxillofac Surg Clin North Am. 2017;25(1):47-54. | Excluded; not a clinical study |
| 475 | Sahm G. [Success and failure in orthodontic treatment following temporomandibular condylar fractures]. Fortschr Kieferorthop. 1988;49(6):557-67. | Excluded; not a clinical study |
| 476 | Stephens RM, Naini FB. A new non-surgical technique for management of high condylar neck fractures. Ann R Coll Surg Engl. 2014;96(6):482-3. | Excluded; not a clinical study |
| 477 | Guo Z, Xu B, Lu L. Meta-analysis of the Mandibular Condylar Fractures Treatment. Journal of China Medical University. 2009;38(9):709-13. | Excluded; review |
| 478 | Cortés Araya J, Argandoña Pozo J, Pantoja Parada R, Encina Moriamez S. Tratamiento funcional en las fracturas condilares mandibulares. Rev dent Chile. 1996;87(1):14-22. | Excluded; case report |
| 479 | Osben Moreno R, Badillo Coloma O, Duarte Meza V, Vidal Molina C. Management of pediatric condylar fractures: Clinical experience in the Hospital Carlos Van Buren, Valparaiso, Chile. International Journal of Oral and Maxillofacial Surgery. 2011;40(10):1032. | Excluded; case report |
| 480 | Hotz RP. Functional jaw orthopedics in the treatment of condylar fractures. Am J Orthod. 1978;73(4):365-77. | Excluded; case report |
| 481 | Bither S, Mahindra U, Halli R, Bakshi M, Kini Y, Shende M, et al. Electromyographic analysis of anterior temporalis and superficial masseter muscles in mandibular angle fractures--a pilot study. Oral Maxillofac Surg. 2012;16(3):299-304. | Excluded; no collum fracture |
| 482 | Chang SP, Yang Y, Shi LQ, Liu YW, Liu Y, Ma Q. Modification of the measurement of the major variables in mandibular condylar fractures: angulation of sidewards displacement and shortening of the height of the ramus. Br J Oral Maxillofac Surg. 2018;56(2):113-9. | Excluded; patients not in growth period |
| 483 | Cuéllar J, Santana J, Núñez C, Villanueva J. Tratamiento quirúrgico o conservador para fracturas de cóndilo mandibular. Medwave. 2018;18(7):e7344-e. | Excluded; patients not in growth period |
| 484 | Eskitaşcıoğlu T, Ozyazgan I, Coruh A, Günay GK, Yontar Y, Altıparmak M. Fractures of the mandible: a 20-year retrospective analysis of 753 patients. Ulus Travma Acil Cerrahi Derg. 2013;19(4):348-56. | Excluded; patients not in growth period |
| 485 | Forouzanfar T, Lobbezoo F, Overgaauw M, de Groot A, Kommers S, van Selms M, et al. Long-term results and complications after treatment of bilateral fractures of the mandibular condyle. Br J Oral Maxillofac Surg. 2013;51(7):634-8. | Excluded; patients not in growth period |
| 486 | Ho SY, Liao HT, Chen CH, Chen YC, Chen YR, Chen CT. The radiographic and functional outcome of bilateral mandibular condylar head fractures: a comparison between open and closed treatment. Ann Plast Surg. 2015;74 Suppl 2:S93-8. | Excluded; patients not in growth period |
| 487 | Devlin, Laverick S, Jones DC. Devlin et al. Open reduction and internal fixation of fractured mandibular condyles by a retromandibular approach: surgical morbidity and informed consent. Br J Oral Maxillofac Surg 2002; 40 : 23-25. British Journal of Oral & Maxillofacial Surgery. 2002;40(5):453-4. | Excluded; patients not in growth period |
| 488 | Silva AP, Sassi FC, Andrade CR. Oral-motor and electromyographic characterization of patients submitted to open a nd closed reductions of mandibular condyle fracture. Codas. 2016;28(5):558-66. | Excluded; patients not in growth period |
| 489 | Talwar RM, Ellis E, 3rd, Throckmorton GS. Adaptations of the masticatory system after bilateral fractures of the mandibular condylar process. J Oral Maxillofac Surg. 1998;56(4):430-9. | Excluded; patients not in growth period |
| 490 | Throckmorton GS, Talwar RM, Ellis E, 3rd. Changes in masticatory patterns after bilateral fracture of the mandibular condylar process. J Oral Maxillofac Surg. 1999;57(5):500-8; discussion 8-9. | Excluded; patients not in growth period |
| 491 | Andersson J, Hallmer F, Eriksson L. Unilateral mandibular condylar fractures: a 31-year follow-up of non-surgical treatment. Int J Oral Maxillofac Surg. 2007;36(4):310-4. | Excluded; mixed sample / treatment |
| 492 | Chang S, Yang Y, Liu Y, Wang J, Zhang W, Ma Q. How Does the Remodeling Capacity of Children Affect the Morphologic Changes of Fractured Mandibular Condylar Processes After Conservative Treatment? J Oral Maxillofac Surg. 2018;76(6):1279.e1-.e7. | Excluded; mixed sample / treatment |
| 493 | Dijkstra PU, Stegenga B, de Bont LGM, Bos RRM. Function impairment and pain after closed treatment of fractures of the mandibular condyle. Journal of Trauma-Injury Infection and Critical Care. 2005;59(2):422-8. | Excluded; mixed sample / treatment |
| 494 | Eulert S, Proff P, Bokan I, Blens T, Gedrange T, Reuther J, et al. Study on treatment of condylar process fractures of the mandible. Annals of Anatomy-Anatomischer Anzeiger. 2007;189(4):377-83. | Excluded; mixed sample / treatment |
| 495 | Hirschfelder U, Müssig D, Zschiesche S, Hirschfelder H. [Functional orthodontic treatment of condylar fractures--a clinical and computerized radiography study]. Fortschr Kieferorthop. 1987;48(6):504-15. | Excluded; mixed sample / treatment |
| 496 | Ingervall B, Lindahl L. Masticatory muscle function in patients treated for condylar fractures of the mandible. Int J Oral Surg. 1980;9(5):359-66. | Excluded; mixed sample / treatment |
| 497 | Kolk A, Neff A. Long-term results of ORIF of condylar head fractures of the mandible: A prospective 5-year follow-up study of small-fragment positional-screw osteosynthesis (SFPSO). Journal of Cranio-Maxillofacial Surgery. 2015;43(4):452-61. | Excluded; mixed sample / treatment |
| 498 | Kondoh T, Hamada Y, Kamei K, Kobayakawa M, Horie A, Iino M, et al. Comparative study of intra-articular irrigation and corticosteroid injection versus closed reduction with intermaxillary fixation for the management of mandibular condyle fractures. Oral Surgery Oral Medicine Oral Pathology Oral Radiology and Endodontology. 2004;98(6):651-6. | Excluded; mixed sample / treatment |
| 499 | Liu CK, Jing CX, Li W, Wang J, Zhou H, Hu M, et al. Observational Study of Surgical Treatment of Sagittal Fractures of Mandibular Condyle. J Craniofac Surg. 2015;26(4):e359-64. | Excluded; mixed sample / treatment |
| 500 | Marker P, Nielsen A, Bastian HL. Fractures of the mandibular condyle. Part 2: Results of treatment of 348 patients. British Journal of Oral & Maxillofacial Surgery. 2000;38(5):422-6. | Excluded; mixed sample / treatment |
| 501 | Nitzan DW, Palla S. "Closed Reduction" Principles Can Manage Diverse Conditions of Temporomandibular Joint Vertical Height Loss: From Displaced Condylar Fractures to Idiopathic Condylar Resorption. J Oral Maxillofac Surg. 2017;75(6):1163.e1-.e20. | Excluded; mixed sample / treatment |
| 502 | Oezmen Y, Mischkowski RA, Lenzen J, Fischbach R. MRI examination of the TMJ and functional results after conservative and surgical treatment of mandibular condyle fractures. International Journal of Oral and Maxillofacial Surgery. 1998;27(1):33-7. | Excluded; mixed sample / treatment |
| 503 | Osben Moreno R, Badillo Coloma O, Duarte Meza V, Vidal Molina C. Mandibular condylar fractures: 12 years experience maxillofacial surgery service, Hospital Carlos Van Buren, Valparaíso, Chile. International Journal of Oral and Maxillofacial Surgery. 2011;40(10):1033. | Excluded; mixed sample / treatment |
| 504 | Rampaso CL, Mattioli TM, de Andrade Sobrinho J, Rapoport A. Evaluation of prevalence in the treatment of mandible condyle fractures. Rev Col Bras Cir. 2012;39(5):373-6. | Excluded; mixed sample / treatment |
| 505 | Tang Y, Wang X, Zhu Y, Sun H, Zhu M. A Comparative evaluation of CBCT outcomes of two closed treatment methods in intracapsular condylar fractures. Oral Surg Oral Med Oral Pathol Oral Radiol. 2017;123(5):e141-e7. | Excluded; mixed sample / treatment |
| 506 | Trost O, Trouilloud P, Malka G. Open reduction and internal fixation of low subcondylar fractures of mandible through high cervical transmasseteric anteroparotid approach. J Oral Maxillofac Surg. 2009;67(11):2446-51. | Excluded; mixed sample / treatment |
| 507 | Demianczuk AN, Verchere C, Phillips JH. The effect on facial growth of pediatric mandibular fractures. J Craniofac Surg. 1999;10(4):323-8. | Excluded; mixed sample / treatment |
| 508 | Hovinga J, Boering G, Stegenga B. Long-term results of nonsurgical management of condylar fractures in children. Int J Oral Maxillofac Surg. 1999;28(6):429-40. | Excluded; mixed sample / treatment |
| 509 | Tanaka N, Uchide N, Suzuki K, Tashiro T, Tomitsuka K, Kimijima Y, et al. Maxillofacial fractures in children. J Craniomaxillofac Surg. 1993;21(7):289-93. | Excluded; mixed sample / treatment |
| 510 | Vesey J, Van Eeden S, Sweet C, Laraway D, Boyle M, Cooper C. Paediatric mandibular fractures over 15 years: A single centre review. International Journal of Oral and Maxillofacial Surgery. 2015;44:e159. | Excluded; mixed sample / treatment |
| 511 | Veras RB, Kriwalsky MS, Eckert AW, Schubert J, Maurer P. Long-term outcomes after treatment of condylar fracture by intraoral access: a functional and radiologic assessment. J Oral Maxillofac Surg. 2007;65(8):1470-6. | Excluded; mixed sample / treatment |
| 512 | Wang BL, He DM, Chi Y, Cai YH, Cai XY, Yang XJ, et al. Factors affecting the outcomes of non-surgical treatment for intracapsular condylar fractures. International Journal of Clinical and Experimental Medicine. 2016;9(6):10847-55. | Excluded; mixed sample / treatment |
| 513 | Xu X, Shi J, Xu B, Dai J, Zhang S. Treatment of mandibular symphyseal fracture combined with dislocated intracapsular condylar fractures. J Craniofac Surg. 2015;26(2):e181-5. | Excluded; mixed sample / treatment |
| 514 | Yang WG, Chen CT, Tsay PK, Chen YR. Functional results of unilateral mandibular condylar process fractures after open and closed treatment. Journal of Trauma-Injury Infection and Critical Care. 2002;52(3):498-503. | Excluded; mixed sample / treatment |
| 515 | Abdullayev E, Rahimov C, Farzaliyev I, Khankishiyev S, Foroughiasl P. Non-surgical treatment of condylar fractures in children. Oral and Maxillofacial Surgery Cases. 2020;6(1). | Excluded; no functional appliance |
| 516 | Boffano P, Roccia F, Schellino E, Baietto F, Gallesio C, Berrone S. Conservative treatment of unilateral displaced condylar fractures in children with mixed dentition. J Craniofac Surg. 2012;23(5):e376-8. | Excluded; no functional appliance |
| 517 | Fordyce AM, Lalani Z, Songra AK, Hildreth AJ, Carton AT, Hawkesford JE. Intermaxillary fixation is not usually necessary to reduce mandibular fractures. Br J Oral Maxillofac Surg. 1999;37(1):52-7. | Excluded; no functional appliance |
| 518 | Hancock A, Gurney B, Walsh S, Collyer J. Outcomes of 112 condylar fractures treated with open reduction and fixation via retromandibular transparotid approach. British Journal of Oral and Maxillofacial Surgery. 2013;51(6):e94-e5. | Excluded; no functional appliance |
| 519 | Rahimov CH, Abdullayev E, Hasanaov E, Farzaliyev I, Mammadova N. Mandibular condylar fractures: The effectiveness of nonsurgical approach in children. International Journal of Oral and Maxillofacial Surgery. 2015;44:e126-e7. | Excluded; no functional appliance |
| 520 | Remi M, Christine MC, Gael P, Soizick P, Joseph-Andre J. Mandibular fractures in children long term results. International Journal of Pediatric Otorhinolaryngology. 2003;67(1):25-30. | Excluded; no functional appliance |
| 521 | Schettler D, Rehrmann A. Long-term results of functional treatment of condylar fractures with the long bridle according to A. Rehrmann. J Maxillofac Surg. 1975;3(1):14-22. | Excluded; no functional appliance |
| 522 | Theologie-Lygidakis N, Chatzidimitriou K, Tzerbos F, Gouzioti A, Iatrou I. Nonsurgical management of condylar fractures in children: A 15-year clinical retrospective study. J Craniomaxillofac Surg. 2016;44(2):85-93. | Excluded; no functional appliance |
| 523 | Vidal Molina C, Osben Moreno R, Badillo Coloma O, Arriola Silva M, Duarte Meza V. Development and management of complications condylar fractures. International Journal of Oral and Maxillofacial Surgery. 2013;42(10):1236. | Excluded; no functional appliance |
| 524 | Kahl B, Fischbach R, Gerlach KL. Temporomandibular joint morphology in children after treatment of condylar fractures with functional appliance therapy: a follow-up study us computed tomography. Dentomaxillofac Radiol. 1995;24(1):37-45. | Included |
| 525 | Kahl B, Gerlach KL. [Functional treatment after condylar fractures with and without an activator]. Fortschr Kieferorthop. 1990;51(6):352-60. | Included |
| 526 | Kahl-Nieke B, Fischbach R, Gerlach KL. CT analysis of temporomandibular joint state in children 5 years after functional treatment of condylar fractures. Int J Oral Maxillofac Surg. 1994;23(6 Pt 1):332-7. | Included |
| 527 | Kahl-Nieke B, Fischbach R. [A critical evaluation of the functional treatment of mandibular neck fractures in children. The results of a spiral computed tomographic follow-up]. Fortschr Kieferorthop. 1995;56(3):157-64. | Included |
| 528 | Kahl-Nieke B, Fischbach R. Condylar restoration after early TMJ fractures and functional appliance therapy. Part I: Remodelling. J Orofac Orthop. 1998;59(3):150-62. | Included |
| 529 | Kahl-Nieke B, Fischbach R. Condylar restoration after early TMJ fractures and functional appliance therapy. Part II: Muscle evaluation. J Orofac Orthop. 1999;60(1):24-38. | Included |
| 530 | Liu CK, Meng FW, Tan XY, Xu J, Liu HW, Liu SX, et al. Clinical and radiological outcomes after treatment of sagittal fracture of mandibular condyle (SFMC) by using occlusal splint in children. Br J Oral Maxillofac Surg. 2014;52(2):144-8. | Included |
| 531 | Strobl H, Emshoff R, Röthler G. Conservative treatment of unilateral condylar fractures in children: a long-term clinical and radiologic follow-up of 55 patients. Int J Oral Maxillofac Surg. 1999 Apr;28(2):95-8. | Included |
| 532 | Zhao YM, Yang J, Bai RC, Ge LH, Zhang Y. A retrospective study of using removable occlusal splint in the treatment of condylar fracture in children. J Craniomaxillofac Surg. 2014 Oct;42(7):1078-82. | Included |

**Supplement 4**. Statistical analysis of the data from Kahl and Gerlach [1990].

A total of 21 patients were included with an average age of 7.7 years (Standard Deviation [SD]=2.6 years; range 3.9 to 13.7 years). The assessed fractures were Type III (n=11; 52%), Type IV (n=4; 19%) and Type V (n=6; 29%). Average mouth opening was 17.6 mm (SD=6.0 mm) before treatment and 48.4 mm (SD=6.7 mm) after follow-up.

Linear regression analysis of the follow-up mouth opening-analysis with initial mouth opening as covariate indicated no statistically significant differences: functional appliance treatment compared to intramaxillary fixation & functional appliance treatment (difference=-4.9 mm; 95% Confidence Interval[CI]=-13.9 to 4.1 mm; P=0.27) and functional appliance treatment compared to intramaxillary fixation & functional exercises (difference=-1.4 mm; 95% CI=-8.7 to 5.8 mm; P=0.68).

Logistic regression analysis found likewise no significant differences in the proportion of patients having a midline deviation: functional appliance treatment compared to intramaxillary fixation & functional appliance treatment (Odds Ratio [OR]=1.5; 95% CI=0.2 to 14.4; P=0.73) and functional appliance treatment compared to intramaxillary fixation & functional exercises (OR=1.3; 95% CI=0.2 to 9.9; P=0.83).

Similar findings were observed at auscultation, where no significant differences existed: functional appliance treatment compared to intramaxillary fixation & functional appliance treatment (OR=3.8; 95% CI=0.3 to 51.4; P=0.32) and functional appliance treatment compared to intramaxillary fixation & functional exercises (OR=5.3; 95% CI=0.4 to 68.9; P=0.21).

No significant effect modification was seen when controlling for patient age and fracture type.

**Supplement 5**. Statistical analysis of the data from Kahl-Nieke and Fischbach (1998).

| **Factor** | **Category** | **Mesiodistal dimension: injured-healthy condyle absolute difference** | | **Anteroposterior dimension: injured-healthy condyle abdolute difference** | | **Mesiodistal dimension: injured-healthy condyle same size (≤10%)** | | **Anteroposterior dimension: injured-healthy condyle same size (≤10%)** | |
| --- | --- | --- | --- | --- | --- | --- | --- | --- | --- |
|  |  | **b (95% CI)** | **P** | **b (95% CI)** | **P** | **OR (95% CI)** | **P** | **OR (95% CI)** | **P** |
| Fracture category | Type I | Reference |  | Reference |  | Reference |  | Reference |  |
|  | Type II | 0.23 (-0.01, 0.48) | 0.06 | -0.01 (-0.17, 0.14) | 0.86 | 0.40 (0.02, 10.02) | 0.58 | 1.00 (-) | - |
|  | Type III | 0 (-0.29, 0.29) | 1.00 | -0.04 (-0.21, 0.14) | 0.68 | 1.00 (-) | - | 5.00 (0.15, 166.59) | 0.37 |
|  | Type IV | 0.31 (0.02, 0.59) | 0.04 | -0.04 (-0.21, 0.14) | 0.68 | 1.00 (-) | - | 5.00 (0.15, 166.59) | 0.37 |
|  | Type V | 0.11 (-0.09, 0.32) | 0.25 | 0.08 (-0.05, 0.20) | 0.21 | 0.10 (0.01, 1.54) | 0.10 | 2.50 (0.16, 38.60) | 0.51 |
|  | Type VI | - |  | - | - | - | - |  |  |
|  |  |  |  |  |  |  |  |  |  |
| Age | Per year | 0 (-0.03, 0.03) | 0.98 | 0.01 (-0.01, 0.02) | 0.25 | 1.05 (0.80, 1.39) | 0.73 | 0.82 (0.56, 1.19) | 0.29 |
|  |  |  |  |  |  |  |  |  |  |
| Follow-up | Per year | 0.04 (-0.01, 0.08) | 0.08 | 0 (-0.03, 0.02) | 0.80 | 0.61 (0.32, 1.16) | 0.13 | 1.38 (0.79, 2.43) | 0.26 |
|  |  |  |  |  |  |  |  |  |  |
| Sex | Female | Reference |  | Reference |  | Reference |  | Reference |  |
|  | Male | -0.21 (-0.36, -0.07) | 0.006 | 0.01 (-0.09, 0.11) | 0.82 | 31.5 (2.35, 422.30) | 0.009 | 0.50 (0.06, 4.00) | 0.51 |
|  |  |  |  |  |  |  |  |  |  |
| Fracture localization | Low | Reference |  | Reference |  | Reference |  | Reference |  |
|  | High | -0.03 (-0.22, 0.15) | 0.70 | 0.06 (-0.04, 0.15) | 0.22 | 0.57 (0.09, 3.64) | 0.55 | 2.70 (0.33, 21.98) | 0.35 |
|  |  |  |  |  |  |  |  |  |  |
| Luxation | No | Reference |  | Reference |  | Reference |  | Reference |  |
|  | Yes | 0.10 (-0.08, 0.27) | 0.26 | 0.06 (-0.04, 0.15) | 0.22 | 0.07 (0.01, 0.68) | 0.02 | 2.70 (0.33, 21.98) | 0.35 |

b, unstandardized regression coefficient; CI, confidence interval; OR, odds ratio.

**Supplement 6**. Statistical analysis of the data from Kahl-Nieke and Fischbach (1999).

| **Factor** | **Category** | **Volume: injured-healthy condyle absolute difference** | | **Bone density: injured-healthy condyle absolute difference** | | **Volume: injured-healthy condyle same size (≤10%)** | | **Bone density: injured-healthy condyle same size (≤10%)** | |
| --- | --- | --- | --- | --- | --- | --- | --- | --- | --- |
|  |  | **b (95% CI)** | **P** | **b (95% CI)** | **P** | **OR (95% CI)** | **P** | **OR (95% CI)** | **P** |
| Fracture category | Type I | Reference |  | Reference |  | Reference |  | Reference |  |
|  | Type II | -0.22 (-1.40, 0.97) | 0.70 | -2.88 (-16.08, 10.31) | 0.65 | 1.00 (-) | - | 1.00 (-) | - |
|  | Type III | 0.08 (-0.69, 0.86) | 0.82 | 7.38 (-1.25, 16.02) | 0.09 | 1.00 (0.05, 18.91) | 1.00 | 1.00 (0.05, 18.91) | 1.00 |
|  | Type IV | 0.03 (-0.86, 0.93) | 0.94 | 0.52 (-9.46, 10.49) | 0.91 | 2.00 (0.08, 51.59) | 0.68 | 2.00 (0.08, 51.59) | 0.68 |
|  | Type V | 0.18 (-0.41, 0.77) | 0.52 | 0.88 (-5.72, 7.48) | 0.78 | 0.67 (0.06, 6.87) | 0.73 | 1.20 (0.13, 11.05) | 0.87 |
|  | Type VI | - | - |  |  |  |  |  |  |
|  |  |  |  |  |  |  |  |  |  |
| Age | Per year | 0.05 (-0.02, 0.12) | 0.14 | 0.01 (-0.87, 0.89) | 0.98 | 0.88 (0.64, 1.20) | 0.41 | 1.12 (0.84, 1.50) | 0.44 |
|  |  |  |  |  |  |  |  |  |  |
| Sex | Female | Reference |  | Reference |  | Reference |  | Reference |  |
|  | Male | 0.15 (-0.30, 0.60) | 0.48 | 1.93 (-3.59, 7.45) | 0.47 | 0.47 (0.07, 3.04) | 0.43 | 0.71 (0.12, 4.32) | 0.71 |
|  |  |  |  |  |  |  |  |  |  |
| Fracture localization | Low | Reference |  | Reference |  | Reference |  | Reference |  |
|  | High | 0.17 (-0.27, 0.62) | 0.43 | 2.86 (-2.56, 8.28) | 0.28 | 0.47 (0.07, 3.04) | 0.43 | 0.71 (0.12, 4.32) | 0.71 |
|  |  |  |  |  |  |  |  |  |  |
| Luxation | No | Reference |  | Reference |  | Reference |  | Reference |  |
|  | Yes | 0.15 (-0.30, 0.60) | 0.49 | -1.12 (-6.67, 4.43) | 0.68 | 0.64 (0.10, 4.10) | 0.64 | 1.00 (0.17, 5.98) | 1.00 |

b, unstandardized regression coefficient; CI, confidence interval; OR, odds ratio.
